# Effects of an e-health intervention ‘iSupport’ for reducing distress of dementia carers: protocol for a randomised controlled trial and feasibility study

**DOI:** 10.1136/bmjopen-2022-064314

**Published:** 2022-09-21

**Authors:** Gill Windle, Greg Flynn, Zoe Hoare, Patricia Masterson-Algar, Kieren Egan, Rhiannon Tudor Edwards, Carys Jones, Aimee Spector, Katherine Algar-Skaife, Gwenllian Hughes, Paul Brocklehurst, Nia Goulden, Debbie Skelhorn, Joshua Stott

**Affiliations:** 1 School of Medical and Health Sciences, Bangor University, Bangor, UK; 2 Department of Computer and Information Science, University of Strathclyde, Glasgow, UK; 3 Department of Clinical, Educational and Health Psychology, University College London (UCL), London, UK; 4 Department of Neuro-medicine and Movement Science (INB), Norwegian University of Science and Technology (NTNU), Trondheim, Norway

**Keywords:** dementia, telemedicine, health economics

## Abstract

**Introduction:**

In the UK, National Health Service (NHS) guidelines recommend that informal carers of people living with dementia should be offered training to help them develop care skills and manage their own physical and mental health. The WHO recommends access to affordable, proven, well-designed, online technologies for education, skills training and support for dementia carers. In response to these recommendations, this multisite randomised controlled trial (RCT) is the first study in the UK to evaluate the clinical and cost-effectiveness of an online support programme developed by the WHO called ‘iSupport for dementia carers’.

**Methods and analysis:**

350 informal carers (age 18+ years) living in Britain who self-identify as experiencing stress and depression will be recruited. They will be randomised to receive ‘iSupport’, or standardised information about caring for someone with dementia (control–comparison). Data will be collected via videoconferencing (eg, Zoom) or telephone interview at baseline, 3 months and 6 months. Intention-to-treat analysis will ascertain effectiveness in the primary outcomes (distress and depression) and combined cost, and quality-adjusted life-year data will be used to assess cost-effectiveness compared with usual care from a public sector and wider societal perspective. A mixed-methods process evaluation with a subgroup of carers in the intervention (~N=50) will explore the barriers and facilitators to implementing ‘iSupport’. A non-randomised feasibility study will adapt ‘iSupport’ for young carers (n=38 participants, age 11–17 years).

**Ethics and dissemination:**

The research plan was scrutinised by National Institute for Health Research reviewers ahead of funding being awarded. Ethical approval was granted by Bangor University’s School of Health and Medical Sciences Academic Ethics Committee, reference number 2021-16915. Dissemination plans include delivering events for stakeholders, social media, a project website, developing policy briefings, presenting at conferences and producing articles for open access publications.

**Trial registration number:**

ISRCTN17420703.

**Strengths and limitations of this study**

- iSupport for dementia carers was developed by experts at the WHO and is based on techniques with proven therapeutic efficacy; consequently, the content is informed by a considerable body of evidence.
- The ‘real world’ application of the randomised controlled trial requires carers to self-identify as experiencing some level of stress or depression, but some may have mild symptoms, limiting the potential for improving these primary outcomes.
- Although the research assistants will be ‘blind’ to the randomisation, a limitation of the study includes being unable to completely blind the participants to their respective allocation (iSupport or information about being a carer).
- Remote data collection and intervention delivery will potentially reach a broader and more diverse range of carers beyond the geographical boundaries often experienced through in-person data collection; however, this could also create challenges for recruiting to target.
- The feasibility study will work with young people to generate valuable information leading to an adapted version of ‘iSupport’ for young carers.

## Introduction

‘Dementia’ is an umbrella term for a cluster of symptoms that characterise neurodegenerative changes, decline and loss of cognitive functioning. Dementia is one of the leading causes of care dependency, disability and death around the world.^1^ The number of people living with dementia is predicted to increase globally, and it is estimated the number of people living with dementia in the UK will increase 80% by 2040.^2^ The limited medical treatments available for people living with dementia mean that in the UK, most people living with dementia are cared for at home,^3^ supported by a family member or friend who often performs care tasks similar to those carried out by paid health or social service providers. The detrimental impact of caregiving on the physical and mental health of informal carers is well documented^4 5^; a meta-analysis found carers were more stressed, depressed and had lower levels of subjective well-being, physical health and self-efficacy than non-carers.^6^

Dementia carers have expressed a need for: (A) relevant information and knowledge; (B) support with the management of care recipients’ functioning, behavioural and psychological symptoms; (C) support with their own physical and mental health; and (D) support regarding their unbalanced social life.^7^ In the face of these significant challenges, Action Area 5 of the WHO’s Global Action Plan on Dementia 2017–2025 prioritises supporting carers, calling for the provision of accessible evidence-based information to improve knowledge and skills and prevent stress and health problems.^8^

To address these challenges, the WHO developed ‘iSupport’, an evidence-informed e-health intervention designed to help dementia carers provide good care and take care of themselves. The content reflects evidence that the most effective interventions for carers’ psychological health should incorporate both an educational component to enhance knowledge and a therapeutic component, such as cognitive–behavioural therapy/cognitive reframing.^9^ Such interventions are often delivered in-person; however, the ongoing COVID-19 pandemic led to reductions, delays and withdrawal of many support services for carers.^10^ Online interventions could be one solution to providing support, negating general accessibility barriers such as carers’ time constraints or needing to travel to receive care and support,^11^ due to their convenience of use, low delivery costs and the ability to negate geographical barriers.^12^ The potential for scalability is also relevant, as few e-health interventions for carers are implemented outside a research setting.^13–15^ However, despite their potential, the evidence base remains limited, and high-quality studies are required to enable definitive conclusions about their effectiveness.^16^ In response, this study aims to contribute to this growing area of healthcare delivery.

‘iSupport’ is in the process of global implementation, and there is research underway in The Netherlands, India and Portugal,^17–19^ but to date, there is no published evidence as to the effectiveness of ‘iSupport’. This will be the first study to examine the effectiveness and cost-effectiveness of a globally targeted e-health intervention in a majority English-speaking population of dementia carers. It will also evaluate the feasibility of adapting ‘iSupport’ for young carers (ages 11–17 years). It is vital that current and future carers have access to education programmes that are tailored to address their particular needs,^20^ as current generic dementia support services are not able to address the specific challenges young carers face.

### Research questions

Are carer distress and depression (primary outcomes) significantly reduced in participants allocated to receive ‘iSupport’ compared with participants allocated to a control–comparison group receiving standardised information about caring for someone with dementia?

Are symptoms of anxiety (secondary outcome) significantly reduced and resilience, relationship quality and dementia knowledge (secondary outcomes) significantly increased in participants allocated to receive ‘iSupport’ compared with participants allocated to the control–comparison group?

3. What are participant and contextual barriers and facilitators to implementation of ‘iSupport’?

4. What potential mechanisms might underpin changes in outcomes from using ‘iSupport’?

5. What is the cost-effectiveness of ‘iSupport’ compared with standardised information about dementia?

6. Is it feasible and acceptable to digitally deliver a refined ‘iSupport’ to young carers?

7. What are the carers’ perspectives of ‘iSupport’ in relation to supporting them in an ongoing or future repeated pandemic such as COVID-19?

## Methods and analysis

### Study design

‘iSupport’ for dementia carers is a multicentre randomised controlled trial (RCT) and feasibility study composed of four workstreams (WS). WS1 will evaluate the effectiveness of ‘iSupport’ (compared with a control–comparison) in reducing carer distress and symptoms of depression (multiple primary outcomes), reductions in anxiety, improvements in resilience, relationship quality and dementia knowledge (secondary outcomes). WS2 (process evaluation) will examine how participants engaged with ‘iSupport’, whether there are any barriers to its uptake, and any perceived benefits for the carer. WS3 (health economic evaluation) will calculate the cost-effectiveness of ‘iSupport’ from a public sector perspective^21^ and from a wider societal perspective. WS4 (feasibility study) will adapt ‘iSupport’ for young carers and assesses the feasibility, acceptability and uptake of conducting a larger trial. Online supplemental file 1 contains the objectives for each workstream. This protocol was developed according to the SPIRIT (2013) checklist.^22^ The study runs for 36 months (1 January 2021–31 December 2023). At the end of their involvement in the study, all participants will receive information about regional support services and a £20 voucher.

10.1136/bmjopen-2022-064314.supp1 Supplementary data

### RCT participant recruitment

Carers living in England, Scotland and Wales will be recruited between December 2021 and January 2023 by researchers working from Bangor University (coordinating centre), University College London or University of Strathclyde (collaborating sites). Researchers will advertise the study through social media; our study partners (Alzheimer Scotland and Carers Trust Wales) and other non-statutory organisations will advertise the study to regional groups through their networks, and the Join Dementia Research^23^ register will be used to identify potential participants (online supplemental file 2). All carers who express an interest in taking part will be sent a consent form, information sheet and be invited to discuss their involvement with a researcher in a one-to-one videoconferencing or phone meeting, when the researcher would also assess their eligibility (table 1).

**Table 1:** Eligibility criteria for the RCT

Inclusion criteria	(1) Adults (age 18+ years) who self-identify as an unpaid carer of a person with dementia who is not living in a full-time care facility, caring at least weekly for at least 6 months.
	(2) Self-identify as experiencing at least some stress, depression or anxiety.
	(3) The care recipient has to have a confirmed diagnosis of dementia through self-report of the carer.
Exclusion criteria	(1) Receiving psychological treatment from a mental health specialist at the time of recruitment.
	(2) Unable to comprehend written English.
	(3) No access to the internet.
	(4) Unable to give informed consent to the trial.
	(5) Have previously used ‘iSupport’ materials in the last 12 months.

A nested internal pilot study at each site will monitor progression criteria over the first 6 months of recruitment. Go/review/stop criterion will be assessed by the study’s independent data monitoring committee, and decisions about the study conduct will be made in consultation with the trial steering committee and the Trial Management Group.

### RCT sample size and randomisation

A meta-analysis reported that technology-based interventions for informal carers of people living with dementia are effective in reducing both depression and burden outcomes.^24^ Consequently, both are important outcomes for carers, and the sample size considers these as multiple primary endpoints at 6 months. The multiple primary endpoint estimator in the R package^25 26^ with power of 90% and significance set to 2.5%, established that 262 participants are required at 6 months to have the potential to detect an effect in at least one of these outcomes. The attrition rate was based on nine dementia intervention studies, where the mean retention rate was 15.33% (range 2%–24%). Accommodating a 25% attrition rate by 6 months, the RCT will recruit and randomise 350 participants. Randomisation uses dynamic allocation to protect against subversion.^27^ This ensures the trial maintains good balance to the allocation ratio of 1:1, both within each stratification variable and overall for the trial. Stratification variables will be site, along with age and gender, previously found to influence the outcome measure of caregiver distress.^28^

RCT ‘iSupport’ intervention iSupport is an internet-based psychoeducation and skills development intervention that can be accessed through a personal computer, tablet or mobile phone. The theoretical underpinnings of ‘iSupport’ are based on person-centred care, recognising that dementia care should reflect the individual’s needs, personality and abilities^29^ and are integrated into the interactive content of ‘iSupport’. The self-care techniques are based on theoretically informed programmes with some evidence for benefits, including psychoeducation, relaxation, behavioural activation, cognitive reframing and problem solving.^30^ Participants will access iSupport in their own homes or a place where they are able to access the internet.

**Figure 1:**
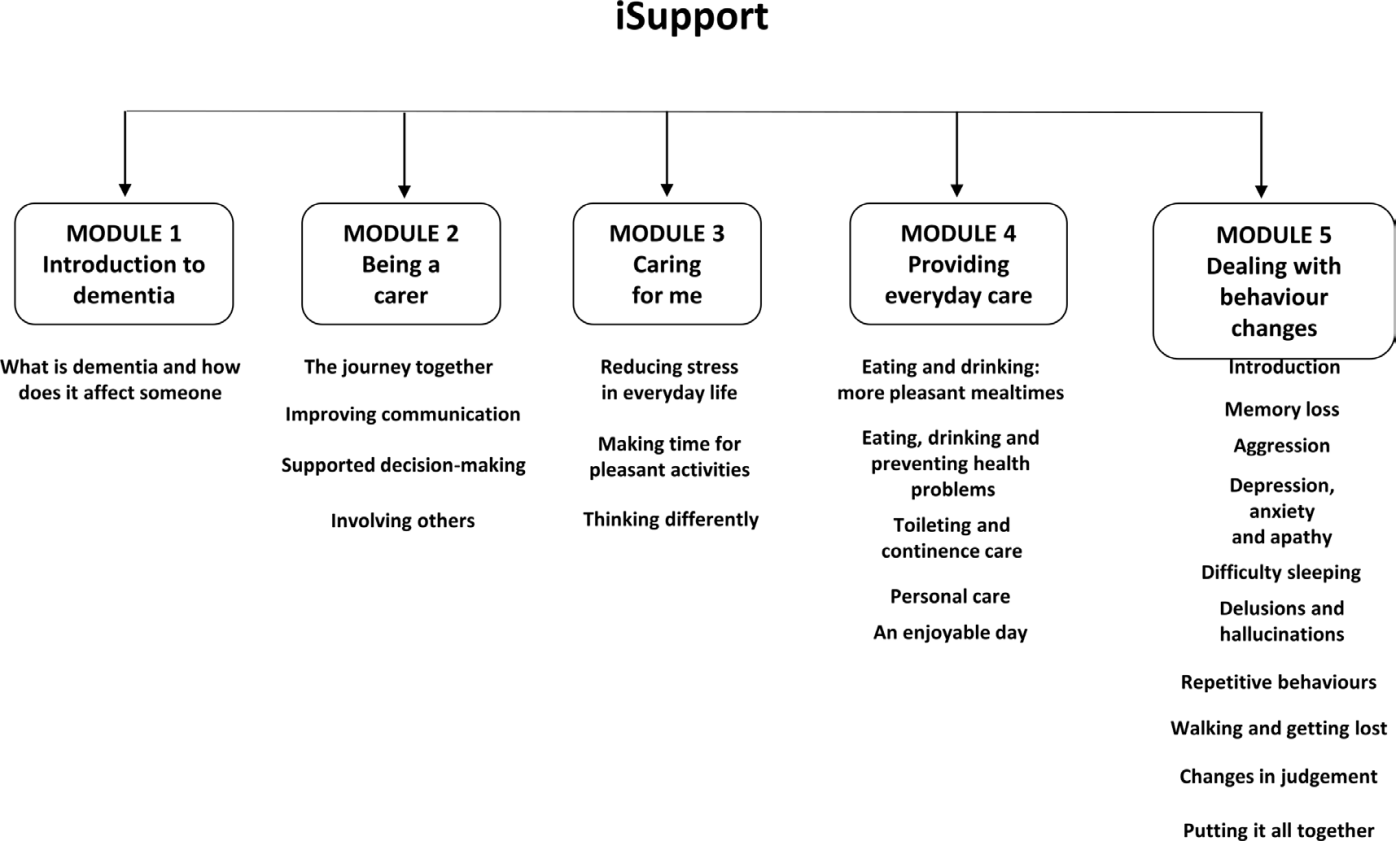
iSupport content.

‘iSupport’ consists of five main themes and 23 accompanying exercises (figure 1). Each exercise takes approximately 5–15 min and follows the same format: information about a topic presented, short interactive exercises and questions with instant feedback on responses, a summary of the lesson and a relaxation exercise. ‘iSupport’ is based on personal choice: carers can construct their own personalised plan and access which sessions they feel are most relevant to them at that point in time. Participants will be advised to use ‘iSupport’ regularly in order to obtain the most benefit. They will be provided with the contact details of an ‘e-coach’ (member of the research team), who will explain anything that is not clear about the ‘iSupport’ programme. The ‘e-coach’ will contact participants allocated to intervention shortly after randomisation, 1 month later and 2 months later (if required by the participant). ‘iSupport’ will be translated into Welsh following WHO adaptation guidelines. Approximately one-fifth of the Welsh population speak Welsh,^31^ and the Welsh Government is committed to offering bilingual services as part of healthcare provision. To improve access, an audio/read aloud function is included in the iSupport programme.

### RCT control–comparison to iSupport

The control–comparison group will receive an information booklet (online and/or hard copy) about caring for someone with dementia, developed by the Alzheimer’s Society.^32^ Alongside this education, carers will receive care-as-usual. They can search for other information or seek help from other providers. Participants allocated to the controlcomparison group will be provided with access to ‘iSupport’ at the end of data collection.

### RCT data collection

**Figure 2:**
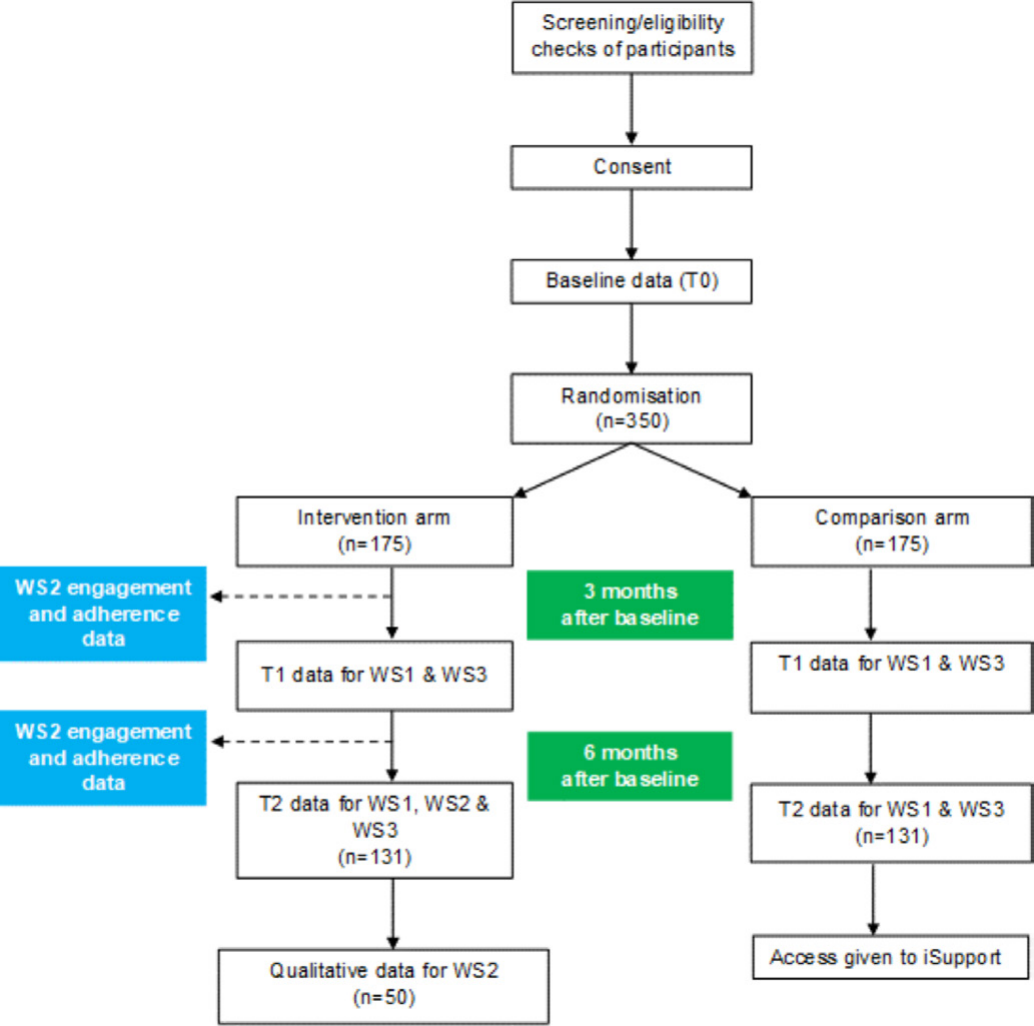
Recruitment flow chart.

Case report forms (CRFs) were initially piloted by researchers, and adjustments made to reduce the time burden to participants without affecting the study’s ability to address the research questions. Data will be collected at three time-points: baseline (T0), 3 months postbaseline (T1 follow-up) and 6 months postbaseline (T2 follow-up). Researchers will interview participants by videoconferencing or phone. Following the baseline interview, researchers will perform the randomisation and the CI, or trial manager will email the participant their group allocation details. Follow-up interviews will be administered by researchers who are blinded to group allocation. An acceptable tolerance for follow-ups will be up to 2 weeks earlier and up to 4 weeks later than the exact T1 or T2 date. Figure 2 shows the flow of the participants through the study.

All data will be entered into an electronic database (MACRO),^33^ and the study statistician will periodically monitor data quality. Table 2 shows the outcome measures, order of administration and the relevance for each workstream.

**Table 2:** Data collection for iSupport RCT

Questionnaire or study-specific questions	Time point	Workstream
Local COVID-19 alert level at date of assessment	T0, T1, T2	1,2,3
Demographic questions	T0	1,2,3
Employment, marital status and living situation questions	T0, T1, T2	1,2,3
12-item Zarit Burden Interview (ZBI-12)^48^*	T0, T1, T2	1,3
10-item Centre for Epidemiological Studies Depression Scale (CES-D-10)*^49^	T0, T1, T2	1,3
EQ-5D-5L^50^	T0, T1, T2	3
Resilience Scale-14^51^	T0, T1, T2	1
Generalised Anxiety Disorder Questionnaire^52^	T0, T1, T2	1
Dementia Knowledge Assessment Scale^53^	T0, T1, T2	1
Adapted Erasumus iMTA informal care questionnaire^54^	T0, T1, T2	3
Service use questions	T0, T1, T2	3
Quality of the Carer-Patient Relationship^55^	T0, T1, T2	1
Dementia Quality of Life – Proxy measure^56^	T0, T1, T2	1,3
Researcher remains blinded to allocation question	T1, T2	1

*Indicates primary outcome measure for WS1.

RCT, randomised controlled trial; WS, workstream.

### WS2 process evaluation sampling and data collection

The process evaluation uses three different approaches to data collection:

1. Semistructured interviews will be undertaken with up to 50 of carers in the intervention group following their T2 interview. The choice of sample size in qualitative research is an area of debate^34^; however, our decision was informed by Ritchie and colleagues,^35^ who recommend that studies employing individual interviews should undertake no more than 50 interviews in order to manage the complexity of the analysis. Baseline data will inform a purposive sampling strategy, and a qualitative sampling matrix will be developed. This matrix will include a diverse range of participant demographic characteristics such as age, gender and caring responsibilities and differences in scores across the the 12-item Zarit Burden Interview (ZBI-12) and the 10-item Centre for Epidemiological Studies Depression Scale (CES-D-10) scores (low, medium and high).

The interview topics will be guided by the process evaluation parameters described in recognised frameworks^36 37^ and drawing on theoretical models such as Normalisation Process Theory.^38^ Motives for declining participation will also be noted where consent is given to understand any barriers to participation and potential selection bias.

1. Data from the online platform will be collected regarding usability (eg, frequency and length of use, which modules/sessions/pages users most frequently visit, average time spend on each module/session/page, whether accessed from tablet, PC or mobile phone). The number of contacts with the e-coach will be recorded.
2. An online evaluation questionnaire will collect quantitative data from all study participants in the intervention arm and will be administered at 6-month follow-up (T2).^39^ This questionnaire will evaluate the overall usability and acceptability of the ‘iSupport’ platform in conjunction with all other data collection methods.

### WS4 feasibility study: participant recruitment

Young carers and professionals who have regular contact with young carers will be recruited through stakeholders’ networks, social media and national carers associations (table 3). Researchers will approach parents or legal guardians of participants under the age of 16 years to explain their child’s involvement and obtain their consent from them. Online supplemental file 3 visualises the phases of the feasibility study.

**Table 3:** Feasibility study eligibility criteria

Inclusion criteria	Young carers (1) Young people between the ages of 11 and 17 years who self-identify as a carer of a person with dementia who is not living in a full-time care facility, caring at least weekly for at least 6 months.	Professionals (1) Have regular contact with young people and young carers (eg, teaching staff involved in pastoral care, young carer charity workers and social workers in children’s services.
	(2) The care recipient has to have a confirmed diagnosis of dementia (through self-report of the carer).	
Exclusion criteria	(1) Receiving treatment from Child and Adolescent Mental Health Services at the time of recruitment.	No regular contact with young people and young carers as part of their work. Unable to comprehend written English. No access to the internet.
	(2) Unable to comprehend written English.	
	(3) No access to the internet.	
	(4) Have previously used ‘iSupport’ materials in the last 12 months.	

### WS4: data collection

#### Phase 1: Adapting ‘iSupport’ for young carers

Three × 3-hour workshops will be conducted either in person or using videoconferencing software (eg, Zoom, Teams or Skype) depending on the government guidelines regarding COVID-19 and safety. At least 2 weeks before the workshops, participants will be given online access to ‘iSupport’ and printed materials for annotations. Workshop 1 will recruit six to eight young carers to discuss their caregiving experiences, which aspects are reflected or missing in ‘iSupport’, and opinions on the content and style of the intervention. Workshop 2 will undertake a similar exercise with six to eight professionals who work with young carers. Feedback will be used to refine ‘iSupport’, which will be shared in workshop 3 with all participants who attended the first two workshops in order to produce a ‘final’ version. Discussions around which outcomes are most important for young carers in relation to ‘iSupport’ will be used to adapt the CRF from the RCT for phase 2.

#### Phase 2: feasibility testing ‘iSupport’ for younger dementia carers

Young carers will test the feasibility of using the refined ‘iSupport’ and following the RCT procedures (except randomisation will not be required). After T2 data collection, participants will complete an online evaluation of their experience using ‘iSupport’. Informed by a methodological framework,^40^ a sample of 30 for phase 2 will provide enough information on the acceptability of the intervention, the appropriateness of data collection forms, the feasibility of recruitment and consent procedures and the most appropriate primary outcome measures.

### Data analysis plans

#### WS1 (research questions 1 and 2)

WS1 primary analysis is an intention-to-treat (ITT) analysis, blinded to treatment allocation. The primary assessment for effectiveness will be adjusted estimates of the ZBI-12 and CES-D-10 scores between the two groups assessed at 6 months. A linear mixed-effects model adjusting for baseline scores, randomising site (random effect) and stratification variables will be fitted for each of the two primary outcomes. Similar models will be fitted for all continuous secondary outcomes. All estimates of effect will be presented together with 95% CIs. The aim is to minimse missing data; however, predictors of missingness will be investigated using regression models, and any predictors found will be considered for inclusion in the models. Multiple imputation will address missing scores where appropriate. Complier Average Causal Effect analysis will assess the impact of the number of times the ‘iSupport’ intervention was accessed. A sensitivity analysis will assess any impact of the outcome measures being completed in Welsh. A full statistical analysis plan will be written and agreed with the independent committees before completion of the data collection.

#### WS2 process evaluation (research questions 3, 4 and 7)

Qualitative interview data analysis will be professionally transcribed verbatim and thematically analysed^41^ using NVivo. Results will also be applied to aspects of the Context and Implementation of Complex Interventions checklist,^42^ which may reflect implementation in a ‘real world’ setting. This analysis will reveal the experiences of using iSupport and its delivery, the barriers and facilitators to its uptake and continued use and the perceived benefits for the carer participating in iSupport and the person they are caring for. Descriptive analyses will profile the System Usability Scale and intervention platform metrics regarding usability (eg, most/least frequently visited pages, the most ‘popular’ modules/sessions).

#### WS3 health economic evaluation (research question 5)

Primary analysis will be an ITT analysis as per WS1. Cost and quality-adjusted life-years data will be combined to calculate an incremental cost-effectiveness ratio. Cost-effectiveness acceptability curves^43^ will show the probability that ‘iSupport’ is cost-effective compared with the control–comparison for a range of willingness-to-pay thresholds. Secondary cost-effectiveness analyses will calculate the cost per unit change in the primary outcome measures. A subgroup analysis will be conducted on the number of times that carers in the intervention group accessed ‘iSupport’. Deterministic sensitivity analyses will be conducted to vary the costs of inputs.

#### WS4 feasibility study (research question 6)

Data from phase 1 workshops will be selectively transcribed, analysed and reported according to established guidance.^44^ All quantitative data collected during phase 2 will be presented descriptively. No inferential testing will be undertaken for this data. The mean change from baseline, associated variances and 95% CIs will be calculated for all selected outcomes. Consideration will be given to the applicability of these outcomes for development into a protocol for a future RCT if the acceptability of the intervention is proven. Success will be defined as acceptability of the recruitment and consent procedure, data collection tools, intervention content and delivery to participants, as well as compliance.

### Patient and public involvement

We involved people living with dementia and their carers in the development of this research. This was achieved by collaborating with the ‘Caban group of dementia educators’, established and supported by the lead applicant’s research centre. The group raised a number of points for the team to consider, with ‘fear of using the internet’ being one area of concern. The group felt a person should be available to help people with iSupport. In response, we built in provision for an ‘e-coach’ to support participants randomised to receive iSupport. Coapplicant Hughes is a young adult carer for her father living with Vascular Dementia and felt the needs of young carers are often overlooked and neglected. She has contributed to the development of this research, especially the conceptualisation of the study design and suggestions for the delivery of WS4, and is assisting with this phase. We will meet with the CABAN group on a regular basis over the study duration, and at a previous meeting, we discussed how a visual participant information sheet could aid recruitment in line with dementia research standards^45^ and that using videoconferencing software would be preferrable to phone calls for arranging and conducting remote interviews. Feedback from this meeting was further referred to when drafting other study materials for consistency.

## Ethics and dissemination

iSupport was granted ethical approval by Bangor University’s School of Medical and Health Sciences Academic Ethics Committee (AEC), reference number 2021-16915. All researchers are fully trained in the study procedures and receive regular supervision. A data management and monitoring plan ensure adherence to the principles of Good Clinical Practice and relevant regulations over the course of the study and to effectively audit the day-to-day conduct at each site. Carers will be provided with clear information and given time to ask questions and consider whether to participate before providing consent (online supplemental file 4). Through the content of our information sheets and consent forms, as well as contact with the research team, participants will understand that they can refuse to participate or withdraw at any time. Changes to the study protocol will be agreed by the funder and an ethics amendment submitted to the Academic Ethics Committee (AEC).

Our research products will include peer-reviewed academic papers, Plain English/Cymraeg Clir summaries of findings, articles for practitioner magazines and a project website. All academic outputs will conform to the reporting procedures in the relevant methodology guidelines (eg, Consolidated Standards of Reporting Trials e-health).^46^ Economic evaluation findings will be reported according to the recently updated Consolidating Health Economic Evaluation Reporting Standards (CHEERS) checklist, highlighting the role of Patient and Public Involvement and Engagement (PPIE) relating to health economics.^47^ We will present at conferences, conduct public and stakeholder events and produce policy briefings.

Our research activities will generate new versions of the iSupport platform for Welsh-language speakers, young carers and a UK-focused version with audio function. If our research shows iSupport is effective, health and care providers, pastoral care teams in schools and charitable organisations will be able to recommend an evidence-based online support service to dementia carers that will be publicly available for use at no cost. We hope this will improve policy and practice around delivering support to dementia carers. For example, UK health and social care could recommend the adapted versions of iSupport in their dementia guidelines. This could reduce demand on community teams at post/diagnosis and initial stages of dementia.

Forthcoming in 2022 in a related project, we will be working in partnership with community organisations to translate and adapt iSupport into three South-Asian languages (Urdu, Punjabi and Bengali) to ensure minority ethnic groups in the UK can also access the support in a way that is culturally appropriate for them.

## Supplementary Material

Reviewer comments

Author's manuscript
