# Supplementary material for: Effects of an e-health intervention ‘iSupport’ for reducing distress of dementia carers: protocol for a randomised controlled trial and feasibility study
Source: BMJ Open. 2022 Sep 21;12(9):e064314. doi: 10.1136/bmjopen-2022-064314 (PMC9494593; doi:10.1136/bmjopen-2022-064314)
Supplement: Supplementary data [file bmjopen-2022-064314supp001.pdf]

Supplementary Material

File 1 Objectives for each work-stream

WS1. A definitive pragmatic individually randomised controlled trial across Wales, Scotland and England, with a six-month nested internal pilot. This will:

• Determine progression of the definitive trial based on a go/review/stop criteria (nested internal pilot).
• Determine the effectiveness of 'iSupport' in reducing symptoms of distress and/or depression.

• Determine the effectiveness of 'iSupport' in reducing symptoms of anxiety.

• Determine the effectiveness of 'iSupport' in improving dementia knowledge, relationship quality and resilience.

• Describe the trial sample according to demographic/socioeconomic characteristics.

WS2. A process evaluation will be conducted in line with the established guidelines for process evaluations of complex evaluations[15,16] to determine the barriers and facilitators to the implementation of 'iSupport' at scale, and the extent it supports carers in the face of the ongoing or future COVID-19 pandemic. This will:
• Determine participant engagement and adherence to 'iSupport'.
• Explore the mechanisms of change.

• Identify the external factors to 'iSupport' which influence the delivery and function of the intervention.

• Explore the contextual factors that influence the scalability of 'iSupport' into wider contexts using the CICI framework.[17]

WS3. A parallel cost-effectiveness analysis, undertaken from both a public sector perspective (NHS, personal social services and local authorities), and a societal perspective (public sector plus opportunity costs). This will:
• Calculate the costs of implementing 'iSupport', including technical support and time spent supporting carers to use the tool.

• Explore patterns of, and estimate the cost of, health and social care resource use for carers in the 'iSupport' and comparison arms of the trial.

• Explore patterns of, and estimate the cost of, health and social care resource use for the care recipients of carers in the trial.

• Explore the opportunity cost of informal care through the measurement of informal care time, types of care task, impacts on carer's leisure and employment hours, and carers' willingness to pay for more support.

• Using QALYs derived from the EQ-5D-5L, determine the cost-effectiveness of 'iSupport' compared to the control condition; conduct secondary cost-effectiveness analyses using the Zarit Burden Interview[18] and the Centre for Epidemiological Studies of Depression Scale (CES-D10).[19,20]

File 2 Recruitment process

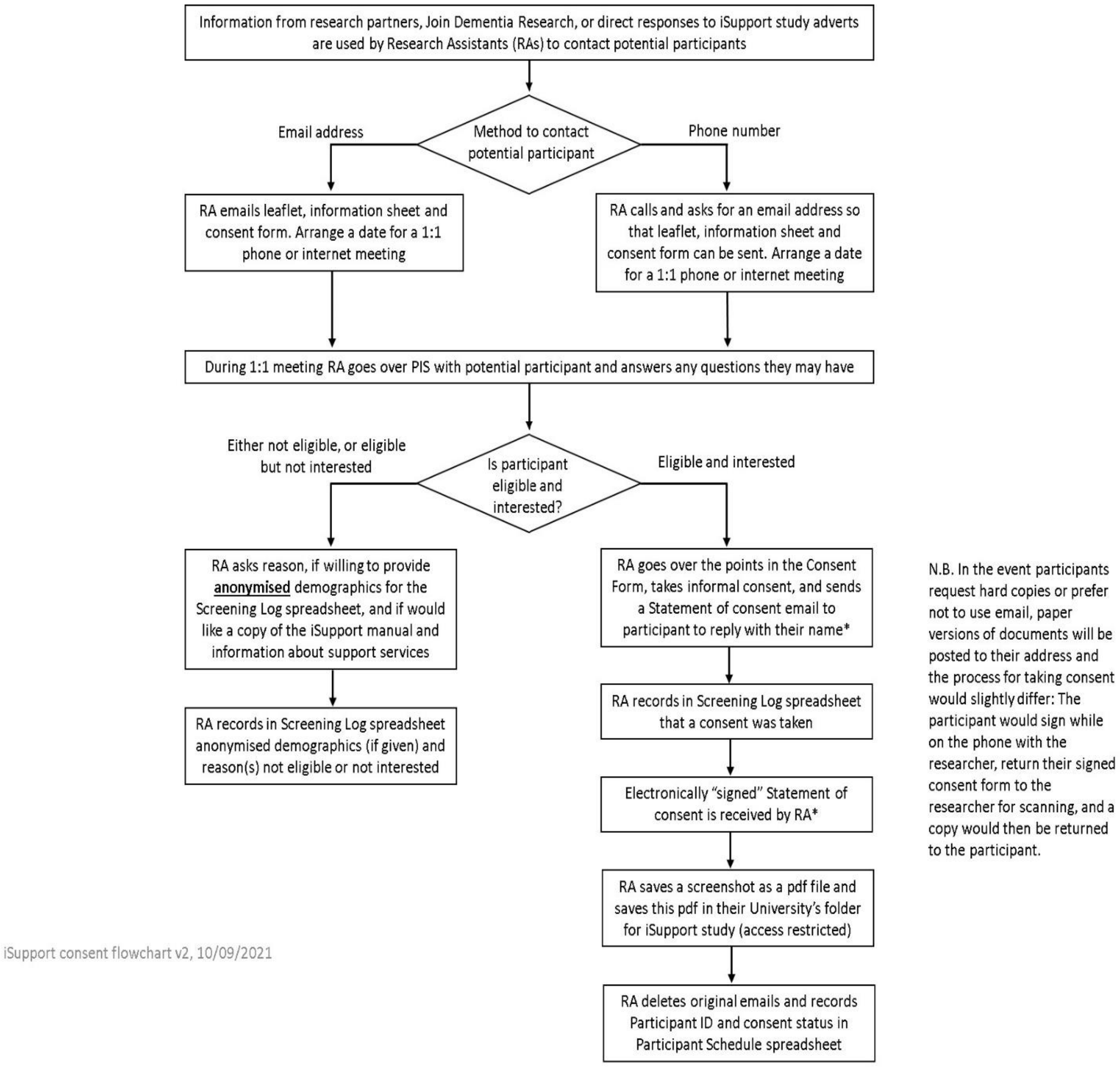

File 3 Feasibility study flowchart

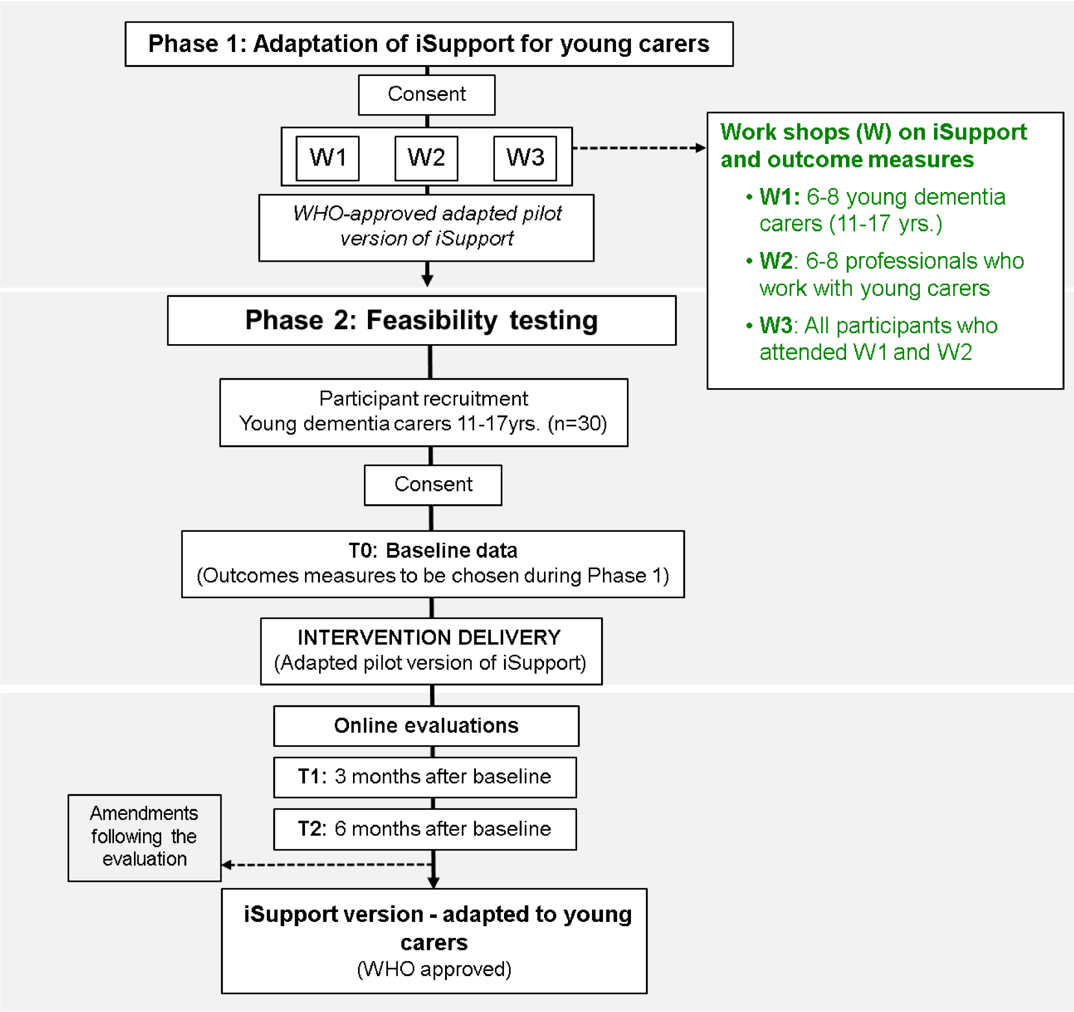

File 4 Consent Forms

iSupport for Dementia Carers – Main trial

Consent Form

Full title of project: A randomised controlled trial and feasibility study of the effects of an e-health intervention 'iSupport' for reducing distress of dementia carers, especially in the ongoing pandemic of COVID-19
Project number: NIHR_130914
Name of lead investigator: Prof. Gill Windle

[The process for technology-mediated consent is detailed in the protocol]

Participant identification number:________________

Please initial each box

1.	I confirm that I have read and understood the information sheet dated 25/10/2021 (version 2) for this study, had the opportunity to ask questions and have had these answered satisfactorily.	
2.	I understand that my participation is voluntary and that I am free to withdraw at any time, without giving any reason. I understand that if I withdraw this will not affect my health care or my legal rights in any way.	
3.	I understand that if I withdraw from the study the research team may continue to use the information that I previously provided up to that point, unless I indicate I do not want them to.	
4.	I understand that the information collected about me may be used to support other research in the future, and may be shared anonymously with other researchers.	
5.	I understand that I will not be identifiable in any data published in relation to this project.	
6.	I understand this study requires my involvement for six months and that I will be contacted by the research team approximately 3 months and 6 months after today's date.	
7.	I understand that if the researchers hear or observe anything that causes serious concern about my health, safety or well-being, or that of another person close to me, they have a duty to inform the lead investigator.	
8.	I agree that my anonymised data can be deposited and securely stored in a data archive.	
9.	I agree to take part in the above study.	

Name of Participant	Date	Signature
________________________	______________	______________
Name of person taking consent	Date	Signature
________________________	______________	______________

Discussing my experiences of using iSupport for Dementia Carers

Consent Form

Full title of project: A randomised controlled trial and feasibility study of the effects of an e-health intervention 'iSupport' for reducing distress of dementia carers, especially in the ongoing pandemic of COVID-19
Project number: NIHR_130914
Name of lead investigator: Prof. Gill Windle

[The process for technology-mediated consent is detailed in the protocol]

Participant identification number:________________

Please initial each box

1.	I confirm that I have read and understood the information sheet dated 25/10/2021 (version 2) for this part of the study, had the opportunity to ask questions and have had these answered satisfactorily.	
2.	I understand that my participation is voluntary and that I am free to withdraw at any time, without giving any reason. I understand that if I withdraw this will not affect my health care or my legal rights in any way.	
3.	I understand that if I withdraw from the study the research team may continue to use the information that I previously provided up to that point, unless I indicate I do not want them to.	
4.	I understand that the information collected about me may be used to support other research in the future, and may be shared anonymously with other researchers.	
5.	I agree to the interview being audio recorded as part of the research study.	
6.	I understand anonymised quotes from the interview may be included in any data published in relation to this project but that I will not be identifiable.	
7.	I understand that if the researchers hear or observe anything that causes serious concern about my health, safety or well-being, or that of another person close to me, they have a duty to inform the lead investigator.	
8.	I agree the anonymised transcripts and audio recordings of the interviews can be deposited and securely stored in a data archive.	
9.	I agree to take part in the above study.	

Name of Participant Date Signature

___________________________ _________________ _________________

Name of person taking consent Date Signature

___________________________ _________________ _________________

Adaptation of iSupport for younger dementia carers (Phase 1) Consent Form

Full title of project: A randomised controlled trial and feasibility study of the effects of an e-health intervention 'iSupport' for reducing distress of dementia carers, especially in the ongoing pandemic of COVID-19
Project number: NIHR_130914
Name of lead investigator: Prof. Gill Windle

[The process for technology-mediated consent is detailed in the protocol]

Participant identification number:________________

Please initial each box

1.	I confirm that I have read and understood the information sheet dated 25/10/2021 (version 3) for this study, had the opportunity to ask questions and have had these answered satisfactorily.	
2.	I understand that my participation is voluntary and that I am free to withdraw at any time, without giving any reason. I understand that if I withdraw this will not affect my health care or my legal rights in any way.	
3.	I agree to the workshops being audio and video recorded by the researcher as part of the study.	
4.	I understand that if I withdraw from the study the research team may continue to use the information that I previously provided up to that point, unless I indicate I do not want them to.	
5.	I understand that the information collected about me may be used to support other research in the future, and may be shared anonymously with other researchers.	
6.	I understand that I will not be identifiable in any data published in relation to this project.	
7.	I agree that my anonymised data can be deposited and securely stored in a data archive.	
8.	I understand that if the researchers hear or observe anything that causes serious concern about my health, safety or well-being, or that of another person close to me, they have a duty to inform the lead investigator.	
9.	I understand that as part of the study there is a procedure in place which deals with disclosures of malpractice or abuse reported by participants and in such instances researchers will be required to break confidentiality.	
10.	I agree to take part in the above study.	

Name of Participant	Date	Signature
________________________	________________	________________
Name of person taking consent	Date	Signature
________________________	________________	________________

Feasibility testing iSupport for younger dementia carers (Phase 2) Consent Form

Full title of project: A randomised controlled trial and feasibility study of the effects of an e-health intervention 'iSupport' for reducing distress of dementia carers, especially in the ongoing pandemic of COVID-19
Project number: NIHR_130914
Name of lead investigator: Prof. Gill Windle

[The process for technology-mediated consent is detailed in the protocol]

Participant identification number:________________

Please initial each box

1.	I confirm that I have read and understood the information sheet dated 25/10/2021 (version 2) for this study, had the opportunity to ask questions and have had these answered satisfactorily.	
2.	I understand that my participation is voluntary and that I am free to withdraw at any time, without giving any reason. I understand that if I withdraw this will not affect my health care or my legal rights in any way.	
3.	I understand this study requires my involvement for six months and that I will be contacted by the research team approximately 3 months and 6 months after today's date.	
4.	I understand that if I withdraw from the study the research team may continue to use the information that I previously provided up to that point, unless I indicate I do not want them to.	
5.	I understand that the information collected about me may be used to support other research in the future, and may be shared anonymously with other researchers.	
6.	I understand that I will not be identifiable in any data published in relation to this project.	
7.	I agree that my anonymised data can be deposited and securely stored in a data archive.	
8.	I understand that if the researchers hear or observe anything that causes serious concern about my health, safety or well-being, or that of another person close to me, they have a duty to inform the lead investigator.	
9.	I understand that as part of the study there is a procedure in place which deals with disclosures of malpractice or abuse reported by participants and in such instances researchers will be required to break confidentiality.	
10.	I agree to take part in the above study.	

Name of Participant	Date	Signature
________________	________________	________________
Name of person taking consent	Date	Signature
________________	________________	________________
