# Supplementary material for: Effects of an e-health intervention ‘iSupport’ for reducing distress of dementia carers: protocol for a randomised controlled trial and feasibility study
Source: BMJ Open. 2022 Sep 21;12(9):e064314. doi: 10.1136/bmjopen-2022-064314 (PMC9494593; doi:10.1136/bmjopen-2022-064314)
Supplement: Author's manuscript [file bmjopen-2022-064314.draft_revisions.pdf]

BMJ Open is committed to open peer review. As part of this commitment we make the peer review history of every article we publish publicly available.

When an article is published we post the peer reviewers' comments and the authors' responses online. We also post the versions of the paper that were used during peer review. These are the versions that the peer review comments apply to.

The versions of the paper that follow are the versions that were submitted during the peer review process. They are not the versions of record or the final published versions. They should not be cited or distributed as the published version of this manuscript.

BMJ Open is an open access journal and the full, final, typeset and author-corrected version of record of the manuscript is available on our site with no access controls, subscription charges or pay-per-view fees (http://bmjopen.bmj.com).

If you have any questions on BMJ Open's open peer review process please email info.bmjopen@bmj.com

BMJ Open

A protocol for a randomised controlled trial and feasibility study of the effects of an e-health intervention 'iSupport' for reducing distress of dementia carers.

Journal:	*BMJ Open*
Manuscript ID	bmjopen-2022-064314
Article Type:	Protocol
Date Submitted by the Author:	14-Jun-2022
Complete List of Authors:	Windle, Gill; Bangor University<br>Flynn, Greg; Bangor University<br>Hoare, Zoe; Bangor University, North Wales Organisation for Randomised Trials in Health<br>Masterson-Algar, Patricia; Bangor University College of Health and Behavioural Sciences, School of Health Sciences<br>Egan, Kieren; University of Strathclyde, Department of Computer and Information Science<br>Edwards, Rhiannon; Bangor University, Centre for Health Economics & Medicines Evaluation<br>Jones, Carys; Bangor Univ<br>Spector, Aimee; University College London (UCL), Department of Clinical, Educational and Health Psychology<br>Algar-Skaife, Katherine; NTNU), Department of Neuro-medicine and Movement Science<br>Hughes, Gwenllian; Bangor University<br>Brocklehurst, P; Bangor University<br>Goulden, Nia; Bangor University, NWORTH CTU<br>Skelhorn, Debbie; Bangor University<br>Stott, Joshua; University College London Research Department of Clinical Educational and Health Psychology
Keywords:	Dementia < NEUROLOGY, Telemedicine < BIOTECHNOLOGY & BIOINFORMATICS, HEALTH ECONOMICS

SCHOLARONE™
Manuscripts

A protocol for a randomised controlled trial and feasibility study of the effects of an e-health intervention 'iSupport' for reducing distress of dementia carers.

Authors

Gill Windle[1], Greg Flynn[1], Zoë Hoare[1], Patricia Masterson-Algar[1], Kieren Egan[2], Rhiannon Tudor Edwards[1], Carys Jones[1], Aimee Spector[3], Katherine Algar-Skaife[4], Gwenllian Hughes[1], Paul Brocklehurst[1], Nia Goulden[1], Debbie Skelhorn[1], Joshua Stott[3].

[1] School of Medical and Health Sciences, Bangor University, Bangor, UK

[2] Department of Computer and Information Science, University of Strathclyde, UK

[3] Department of Clinical, Educational and Health Psychology, University College London (UCL), London, UK

[4] Department of Neuro-medicine and Movement Science (INB), Norwegian University of Science and Technology (NTNU), Trondheim, Norway.

Corresponding author: Professor Gill Windle, DSDC Wales Research Centre/Ageing and Dementia @ Bangor, School of Medical and Health Sciences, Bangor University, Fron Heulog, Bangor, Gwynedd, Wales. g.windle@bangor.ac.uk

Word count for article summary and main text =3588 (excluding title page, abstract, references, figures and tables).

For peer review only - http://bmjopen.bmj.com/site/about/guidelines.xhtml

ABSTRACT

Introduction: In the United Kingdom, National Health Service (NHS) guidelines recommend that informal carers of people living with dementia should be offered training to help them develop care skills and manage their own physical and mental health. The World Health Organization (WHO) recommend access to affordable, proven, well-designed, online technologies for education, skills-training and support for dementia carers. In response to these recommendations, this multi-site randomised controlled trial (RCT) is the first study in the UK to evaluate the clinical and cost-effectiveness of an online support programme developed by the WHO called 'iSupport for dementia carers.'

Methods and analysis: 350 informal carers (age 18+) living in Britain who self-identify as experiencing stress and depression will be recruited. They will be randomised to receive 'iSupport', or standardised information about caring for someone with dementia (control-comparison). Data will be collected via videoconferencing (e.g. Zoom) or telephone interview at baseline, three months and six months. Intention-to-treat analysis will ascertain effectiveness in the primary outcomes (distress and depression) and combined cost and quality adjusted life year (QALY) data will be used to assess cost-effectiveness compared with usual care from a public sector and wider societal perspective. A mixed-methods process evaluation with a sub-group of carers in the intervention (~N=50) will explore the barriers and facilitators to implementing 'iSupport'. A non-randomised feasibility study will adapt 'iSupport' for young carers (N=38 participants, age 11-17).

For peer review only - http://bmjopen.bmj.com/site/about/guidelines.xhtml

Ethics and dissemination: The research plan was scrutinised by National Institute for Health Research reviewers ahead of funding being awarded. Ethical approval was granted by Bangor University's School of Health and Medical Sciences Academic Ethics Committee, reference number 2021-16915. Dissemination plans include delivering events for stakeholders, social media, a project website, developing policy briefings, presenting at conferences and producing articles for open access publications.

Registration details: ISRCTN reference number17420703.

Keywords: dementia; carers; iSupport; technology; internet; website; randomized controlled trial; protocol; process evaluation; cost effectiveness; young carers.

For peer review only

ARTICLE SUMMARY

Strengths and limitations of this study

- iSupport for dementia carers was developed by experts at the WHO and is based on techniques with proven therapeutic efficacy, consequently the content is informed by a considerable body of evidence.
- The 'real world' application of the RCT requires carers to self-identify as experiencing some level of stress or depression, but some may have mild symptoms, limiting the potential for improving these primary outcomes.
- Although the research assistants will be 'blind' to the randomisation, a limitation of the study includes being unable to completely blind the participants to their respective allocation (iSupport or information about being a carer).
- Remote data collection and intervention delivery will potentially reach a broader and more diverse range of carers beyond the geographical boundaries often experienced through in-person data collection, however this could also create challenges for recruiting to target.
- The feasibility study will work with young people to generate valuable information leading to an adapted version of 'iSupport' for young carers.

For peer review only

INTRODUCTION

'Dementia' is an umbrella term for a cluster of symptoms that characterise neurodegenerative changes, decline and loss of cognitive functioning. Dementia is one of the leading causes of care dependency, disability and death around the world.[1] The number of people living with dementia is predicted to increase globally, and it is estimated the number of people living with dementia in the UK will increase 80% by 2040.[2] The limited medical treatments available for people living with dementia mean that in the UK, most people living with dementia are cared for at home,[3] supported by a family member or friend who often performs care tasks similar to those carried out by paid health or social service providers. The detrimental impact of caregiving on the physical and mental health of informal carers is well-documented;[4,5] a meta-analysis found carers were more stressed, depressed, and had lower levels of subjective well-being, physical health, and self-efficacy than non-carers.[6]

Dementia carers have expressed a need for: a) relevant information and knowledge; b) support with the management of care recipients' functioning, behavioural and psychological symptoms; c) support with their own physical and mental health; d) support regarding their unbalanced social life.[7] In the face of these significant challenges, Action Area 5 of the World Health Organization's Global Action Plan on Dementia 2017-2025 prioritises supporting carers, calling for the provision of accessible evidence-based information to improve knowledge and skills, and prevent stress and health problems.[8]

To address these challenges the World Health Organization (WHO) developed 'iSupport', an evidence-informed e-health intervention designed to help dementia carers provide good care and take care of themselves. The content reflects evidence that the most effective interventions for carers' psychological health should incorporate both an educational

For peer review only - http://bmjopen.bmj.com/site/about/guidelines.xhtml

component to enhance knowledge, and a therapeutic component, such as Cognitive Behavioural Therapy /cognitive reframing.[9] Such interventions are often delivered in-person, however the ongoing Covid-19 pandemic led to reductions, delays and withdrawal of many support services for carers.[10] Online interventions could be one solution to providing support, negating general accessibility barriers such as carers' time constraints or needing to travel to receive care and support,[11] due to their convenience of use, low delivery costs, and the ability to negate geographical barriers.[12] The potential for scalability is also relevant, as few e-health interventions for carers are implemented outside a research setting.[13,14,15] However, despite their potential, the evidence base remains limited and high-quality studies are required to enable definitive conclusions about their effectiveness.[16] In response, this study aims to contribute to this growing area of healthcare delivery.

'iSupport' is in the process of global implementation and there is research underway in The Netherlands, India and Portugal,[17,18,19] but to date, there is no published evidence as to the effectiveness of 'iSupport'. This will be the first study to examine the effectiveness and cost effectiveness of a globally targeted e-health intervention in a majority English-speaking population of dementia carers. It will also evaluate the feasibility of adapting 'iSupport' for young carers (ages 11-17). It is vital that current and future carers have access to education programmes that are tailored to address their particular needs,[20] as current generic dementia support services are not able to address the specific challenges young carers face.

Research questions

1. Are carer distress and depression (primary outcomes) significantly reduced in participants allocated to receive 'iSupport' compared to participants allocated to a control-comparison group receiving standardised information about caring for someone with dementia?

For peer review only - http://bmjopen.bmj.com/site/about/guidelines.xhtml

2. Are symptoms of anxiety (secondary outcome) significantly reduced and resilience, relationship quality and dementia knowledge (secondary outcomes) significantly increased in participants allocated to receive 'iSupport' compared to participants allocated to the control-comparison group?

3. What are participant and contextual barriers and facilitators to implementation of 'iSupport'?

4. What potential mechanisms might underpin changes in outcomes from using 'iSupport'?

5. What is the cost-effectiveness of 'iSupport' compared to standardised information about dementia?

6. Is it feasible and acceptable to digitally deliver a refined 'iSupport' to young carers?

7. What are the carers' perspectives of 'iSupport' in relation to supporting them in an ongoing or future repeated pandemic such as COVID-19?

METHODS AND ANALYSIS

Study design

'iSupport' for dementia carers is a multi-centre randomised controlled trial (RCT) and feasibility study composed of four workstreams (WS). WS1 will evaluate the effectiveness of 'iSupport' (compared to a control-comparison) in reducing carer distress and symptoms of depression (multiple primary outcomes), reductions in anxiety, improvements in resilience, relationship quality, and dementia knowledge (secondary outcomes). WS2 (process evaluation) will examine how participants engaged with 'iSupport', whether there are any barriers to its uptake, and any perceived benefits for the carer. WS3 (health economic evaluation) will calculate the cost-effectiveness of 'iSupport' from a public sector perspective[21] and from a wider societal perspective. WS4 (feasibility study) will adapt 'iSupport' for young carers, and assesses the feasibility, acceptability and uptake of conducting a larger trial. Supplementary File 1 contains the objectives for each workstream. This protocol was developed according to the SPIRIT (2013) checklist[22]. The study runs for

For peer review only - http://bmjopen.bmj.com/site/about/guidelines.xhtml

36 months (1st January 2021 to 31st December 2023). At the end of their involvement in the study, all participants will receive information about regional support services and a £20 voucher.

RCT participant recruitment

Carers living in England, Scotland and Wales will be recruited between December 2021 and January 2023 by researchers working from Bangor University (co-ordinating centre), University College London, or University of Strathclyde (collaborating sites). Researchers will advertise the study through social media; our study partners (Alzheimer Scotland and Carers Trust Wales) and other non-statutory organisations will advertise the study to regional groups through their networks; and the Join Dementia Research (JDR)[23] register will be used to identify potential participants (Supplementary File 2). All carers who express an interest in taking part will be sent a consent form, information sheet and be invited to discuss their involvement with a researcher in a one-to-one videoconferencing or phone meeting, when the researcher would also assess their eligibility (Table 1).

Table 1: Eligibility criteria for the RCT.

Inclusion criteria	1) Adults (age 18+) who self-identify as an unpaid carer of a person with dementia who is not living in a full-time care facility, caring at least weekly for at least 6 months.<br>2) Self-identify as experiencing at least some stress, depression or anxiety.<br>3) The care recipient has to have a confirmed diagnosis of dementia through self-report of the carer.
Exclusion criteria	1) Receiving psychological treatment from a mental health specialist at the time of recruitment.

For peer review only

2) Unable to comprehend written English.

3) No access to the internet.

4) Unable to give informed consent to the trial.

5) Have previously used ‘iSupport’ materials in the last 12 months.

A nested internal pilot study at each site will monitor progression criteria over the first six months of recruitment. Go/review/stop criterion will be assessed by the study’s independent data monitoring committee (IDMC), and decisions about the study conduct will be made in consultation with the trial steering committee (TSC) and the Trial Management Group (TMG).

For peer review only

RCT sample size and randomisation

A meta-analysis reported that technology-based interventions for informal carers of people living with dementia are effective in reducing both depression and burden outcomes.[24] Consequently both are important outcomes for carers, and the sample size considers these as multiple primary endpoints at six months. The multiple primary endpoint estimator in the R package [25,26] with power of 90% and significance set to 2.5%, established that 262 participants are required at six months to have the potential to detect an effect in at least one of these outcomes. The attrition rate was based on 9 dementia intervention studies, where the mean retention rate was 15.33% (range 2%-24%). Accommodating a 25% attrition rate by six months, the RCT will recruit and randomise 350 participants. Randomisation uses dynamic allocation to protect against subversion.[27] This ensures the trial maintains good balance to the allocation ratio of 1:1, both within each stratification variable and overall for the trial. Stratification variables will be site, along with age and gender, previously found to influence the outcome measure of caregiver distress.[28]

For peer review only - http://bmjopen.bmj.com/site/about/guidelines.xhtml

RCT ‘iSupport’ intervention

iSupport is an internet-based psychoeducation and skills development intervention that can be accessed through a personal computer, tablet or mobile phone. The theoretical underpinnings of ‘iSupport’ are based on person-centred care, recognising that dementia care should reflect the individual’s needs, personality and abilities[29] and are integrated into the interactive content of ‘iSupport’. The self-care techniques are based on theoretically informed programmes with some evidence for benefits, including psychoeducation, relaxation, behavioural activation, cognitive reframing, and problem-solving.[30] Participants will access iSupport in their own homes or a place where they are able to access the internet.

‘iSupport’ consists of five main themes and twenty-three accompanying exercises (Figure 1). Each exercise takes approximately 5-15 minutes and follows the same format: information about a topic presented; short interactive exercises and questions with instant feedback on responses; a summary of the lesson; a relaxation exercise. ‘iSupport’ is based on personal choice: carers can construct their own personalised plan and access which sessions they feel are most relevant to them at that point in time. Participants will be advised to use ‘iSupport’ regularly in order to obtain the most benefit. They will be provided with the contact details of an ‘e-coach’ (member of the research team), who will explain anything that is not clear about the ‘iSupport’ programme. The ‘e-coach’ will contact participants allocated to intervention shortly after randomisation, 1 month later and 2 months later (if required by the participant). ‘iSupport’ will be translated into Welsh following WHO adaptation guidelines. Approximately one-fifth of the Welsh population speak Welsh[31] and the Welsh Government is committed to offering bilingual services as part of health care provision. To improve access, an audio/read aloud function is included in the iSupport programme.

(Figure 1)

For peer review only - http://bmjopen.bmj.com/site/about/guidelines.xhtml

RCT control-comparison to iSupport

The control-comparison group will receive an information booklet (online and/or hard copy) about caring for someone with dementia, developed by the Alzheimer's Society.[32] Alongside this education, carers will receive care-as-usual. They can search for other information or seek help from other providers. Participants allocated to the control-comparison group will be provided with access to 'iSupport' at the end of data collection**.**

RCT data collection

Case report forms (CRFs) were initially piloted by researchers, and adjustments made to reduce the time-burden to participants without affecting the study's ability to address the research questions. Data will be collected at three time-points: Baseline (T0), 3-months post-baseline (T1 follow-up), and 6-months post-baseline (T2 follow-up). Researchers will interview participants by videoconferencing or phone. Following the baseline interview, researchers will perform the randomisation and the CI or trial manager will email the participant their group allocation details. Follow-up interviews will be administered by researchers who are blinded to group allocation. An acceptable tolerance for follow-ups will be up to 2 weeks earlier and up to 4 weeks later than the exact T1 or T2 date. Figure 2 shows the flow of the participants through the study.

(Figure 2)

All data will be entered into an electronic database (MACRO),[33] and the study statistician will periodically monitor data quality. Table 2 shows the outcome measures, order of administration and the relevance for each workstream.

For peer review only

Table 2: Data collection for iSupport RCT

Questionnaire or study-specific questions	Time point	Workstream
Local COVID-19 alert level at date of assessment	T0, T1, T2	1,2,3
Demographic questions	T0	1,2,3
Employment, marital status, and living situation questions	T0, T1, T2	1,2,3
12-item Zarit Burden Interview (ZBI-12)*[i]	T0, T1, T2	1,3
10-item Centre for Epidemiological Studies Depression Scale (CES-D-10)*[ii]	T0, T1, T2	1,3
EQ-5D-5L[iii]	T0, T1, T2	3
Resilience Scale-14 (RS-14)[iv]	T0, T1, T2	1
Generalised Anxiety Disorder Questionnaire (GAD-7)[v]	T0, T1, T2	1
Dementia Knowledge Assessment Scale (DKAS)[vi]	T0, T1, T2	1
Adapted Erasumus iMTA informal care questionnaire[vii]	T0, T1, T2	3
Service use questions	T0, T1, T2	3
Quality of the Carer-Patient Relationship (QCPR)[viii]	T0, T1, T2	1
Dementia Quality of Life - Proxy measure (DEMQOL-Proxy)[ix]	T0, T1, T2	1,3
Researcher remains blinded to allocation question	T1, T2	1

*indicates primary outcome measure for WS1

WS2 Process evaluation sampling and data collection

The process evaluation utilises three different approaches to data collection:

1. Semi-structured interviews will be undertaken with up to 50 of carers in the intervention group following their T2 interview. The choice of sample size in qualitative research is an area of debate,[34] however our decision was informed by Ritchie and colleagues[35] who recommend that studies employing individual interviews should undertake no more than 50

For peer review only - http://bmjopen.bmj.com/site/about/guidelines.xhtml

interviews in order to manage the complexity of the analysis. Baseline data will inform a purposive sampling strategy and a qualitative sampling matrix will be developed. This matrix will include a diverse range of participant demographic characteristics such as age, gender and caring responsibilities and differences in scores across the the ZBI-12 and the CES-D-10 scores (low, medium, high).

The interview topics will be guided by the process evaluation parameters described in recognised frameworks,[36,37] and drawing upon theoretical models such as Normalisation Process Theory (NPT).[38] Motives for declining participation will also be noted where consent is given, to understand any barriers to participation and potential selection bias.

2. Data from the online platform will be collected regarding usability (e.g. frequency and length of use, which modules/sessions/pages users most frequently visit, average time spend on each module/session/page, whether accessed from tablet, PC or mobile phone). The number of contacts with the e-coach will be recorded.

3. An online evaluation questionnaire will collect quantitative data from all study participants in the intervention arm and will be administered at 6-month follow-up (T2). This questionnaire will evaluate the overall usability and acceptability of the 'iSupport' platform in conjunction with all other data collection methods.

WS4 Feasibility study: participant recruitment

Young carers and professionals who have regular contact with young carers will be recruited through stakeholders' networks, social media, and national carers associations (Table 3). Researchers will approach parents or legal guardians of participants under the age of 16 to

For peer review only - http://bmjopen.bmj.com/site/about/guidelines.xhtml

explain their child's involvement and obtain their consent from them. Supplementary File 3 visualises the phases of the feasibility study.

Table 3: Feasibility study eligibility criteria

	**Young carers**	**Professionals**
Inclusion criteria	1) Young people between the ages of 11 - 17 who self-identify as a carer of a person with dementia who is not living in a full-time care facility, caring at least weekly for at least 6 months.<br>2) The care recipient has to have a confirmed diagnosis of dementia (through self-report of the carer).	1) Have regular contact with young people and young carers (e.g. teaching staff involved in pastoral care, young carer charity workers, social workers in children's services.
Exclusion criteria	1) Receiving treatment from Child and Adolescent Mental Health Services (CAMHS) at the time of recruitment.<br>2) Unable to comprehend written English.<br>3) No access to the internet.<br>4) Have previously used 'iSupport' materials in the last 12 months.	1) No regular contact with young people and young carers as part of their work.<br>2) Unable to comprehend written English.<br>3) No access to the internet.

For peer review only - http://bmjopen.bmj.com/site/about/guidelines.xhtml

WS4: data collection

Phase 1: Adapting 'iSupport' for young carers

Three x 3-hour workshops will be conducted either in person or using videoconferencing software (e.g. Zoom, Teams or Skype) depending on the government guidelines regarding COVID-19 and safety. At least two weeks before the workshops, participants will be given online access to 'iSupport' and printed materials for annotations. Workshop One will recruit 6-8 young carers to discuss their care-giving experiences, which aspects are reflected or missing in 'iSupport', and opinions on the content and style of the intervention. Workshop Two will undertake a similar exercise with 6-8 professionals who work with young carers. Feedback will be used to refine 'iSupport', which will be shared in Workshop Three with all participants who attended the first two workshops in order to produce a "final" version. Discussions around which outcomes are most important for young carers in relation to 'iSupport' will be used to adapt the CRF from the RCT for Phase 2.

Phase 2: Feasibility testing 'iSupport' for younger dementia carers

Young carers will test the feasibility of using the refined 'iSupport' and following the RCT procedures (except randomisation will not be required). After T2 data collection, participants will complete an online evaluation of their experience using 'iSupport'. Informed by a methodological framework,[40] a sample of n=30 for phase 2 will provide enough information on the acceptability of the intervention, the appropriateness of data collection forms, the feasibility of recruitment and consent procedures, and the most appropriate primary outcome measures.

For peer review only - http://bmjopen.bmj.com/site/about/guidelines.xhtml

Data analysis plans

WS1 (research questions 1 and 2).

WS1 Primary analysis is an intention-to-treat (ITT) analysis, blinded to treatment allocation. The primary assessment for effectiveness will be adjusted estimates of the ZBI-12 and CES-D-10 scores between the two groups assessed at 6 months. A linear mixed-effects model adjusting for baseline scores, randomising site (random effect), and stratification variables will be fitted for each of the two primary outcomes. Similar models will be fitted for all continuous secondary outcomes. All estimates of effect will be presented together with 95% confidence intervals. The aim is to minimse missing data; however, predictors of missingness will be investigated using regression models and any predictors found will be considered for inclusion in the models. Multiple imputation will address missing scores where appropriate. Complier Average Causal Effect (CACE) analysis will assess the impact of the number of times the 'iSupport' intervention was accessed. A sensitivity analysis will assess any impact of the outcome measures being completed in Welsh. A full statistical analysis plan will be written and agreed with the independent committees before completion of the data collection.

WS2 Process evaluation (research questions 3, 4 and 7).

Qualitative interview data analysis will be professionally transcribed verbatim and thematically analysed[41] using NVivo. Results will also be applied to aspects of the Context and Implementation of Complex Interventions (CICI) checklist[42], which may reflect implementation in a 'real world' setting. This analysis will reveal the experiences of using iSupport and its delivery, the barriers and facilitators to its uptake and continued use, and the perceived benefits for the carer participating in iSupport and the person they are caring for. Descriptive analyses will profile the System Usability Scale and intervention platform

For peer review only - http://bmjopen.bmj.com/site/about/guidelines.xhtml

metrics regarding usability (e.g. most/least frequently visited pages, the most ‘popular’ modules/sessions).

WS3 Health economic evaluation (research question 5).

Primary analysis will be an ITT analysis as per WS1. Cost and quality-adjusted life years data will be combined to calculate an incremental cost-effectiveness ratio. Cost-effectiveness acceptability curves[43] will show the probability that ‘iSupport’ is cost-effective compared to the control-comparison for a range of willingness-to-pay thresholds. Secondary cost-effectiveness analyses will calculate the cost per unit change in the primary outcome measures. A sub-group analysis will be conducted on the number of times that carers in the intervention group accessed ‘iSupport’. Deterministic sensitivity analyses will be conducted to vary the costs of inputs.

WS4 feasibility study (research question 6).

Data from Phase 1 workshops will be selectively transcribed, analysed and reported according to established guidance[44]. All quantitative data collected during Phase 2 will be presented descriptively. No inferential testing will be undertaken for this data. The mean change from baseline, associated variances, and 95% confidence intervals will be calculated for all selected outcomes. Consideration will be given to the applicability of these outcomes for development into a protocol for a future RCT if the acceptability of the intervention is proven. Success will be defined as acceptability of the recruitment and consent procedure, data collection tools, intervention content and delivery to participants, as well as compliance.

Patient and public involvement statement

We involved people living with dementia and their carers in the development of this research. This was achieved by collaborating with the ‘Caban group of dementia educators’,

For peer review only - http://bmjopen.bmj.com/site/about/guidelines.xhtml

established and supported by the lead applicant's research centre. The group raised a number of points for the team to consider, with 'fear of using the internet' being one area of concern. The group felt a person should be available to help people with iSupport. In response we built in provision for an 'e-coach' to support participants randomised to receive iSupport. Co-applicant Hughes is a young adult carer for her father living with Vascular Dementia and felt the needs of young carers are often overlooked and neglected. She has contributed to the development of this research, especially the conceptualisation of the study design and suggestions for the delivery of WS4, and is assisting with this phase. We will meet with the CABAN group on a regular basis over the study duration, and at a previous meeting we discussed how a visual participant information sheet could aid recruitment in line with dementia research standards,[45] and that using videoconferencing software would be preferrable to phone calls for arranging and conducting remote interviews. Feedback from this meeting was further referred to when drafting other study materials for consistency.

ETHICS AND DISSEMINATION

iSupport was granted ethical approval by Bangor University's School of Medical and Health Sciences Academic Ethics Committee (AEC), reference number 2021-16915. All researchers are fully trained in the study procedures and receive regular supervision. A data management and monitoring plan ensure adherence to the principles of Good Clinical Practice and relevant regulations over the course of the study, and to effectively audit the day-to-day conduct at each site. Carers will be provided with clear information and given time to ask questions and consider whether to participate before providing consent. Through the content of our information sheets and consent forms, as well as contact with the research team, participants will understand that they can refuse to participate or withdraw at any time. Changes to the study protocol will be agreed by the funder and an ethics amendment submitted to the AEC.

For peer review only - http://bmjopen.bmj.com/site/about/guidelines.xhtml

Our research products will include peer-reviewed academic papers, Plain English/Cymraeg Clir summaries of findings, articles for practitioner magazines and a project website. All academic outputs will conform to the reporting procedures in the relevant methodology guidelines (e.g. CONSORT e-health[46]). Economic evaluation findings will be reported according to the recently updated CHEERS checklist, highlighting the role of PPIE relating to health economics.[47] We will present at conferences, conduct public and stakeholder events, and produce policy briefings.

Our research activities will generate new versions of the iSupport platform for Welsh-language speakers, young carers, and a UK-focused version with audio function. If our research shows iSupport is effective, health and care providers, pastoral care teams in schools, and charitable organisations will be able to recommend an evidence-based online support service to dementia carers that will be publicly available for use at no cost. We hope this will improve policy and practice around delivering support to dementia carers. For example UK health and social care could recommend the adapted versions of iSupport in their dementia guidelines. This could reduce demand on community teams at post/diagnosis and initial stages of dementia.

Forthcoming in 2022 in a related project, we will be working in partnership with community organisations to translate and adapt iSupport into three South-Asian languages (Urdu, Punjabi and Bengali) to ensure minority ethnic groups in the UK can also access the support in a way that is culturally appropriate for them.

For peer review only - http://bmjopen.bmj.com/site/about/guidelines.xhtml

AUTHOR CONTRIBUTIONS

GW leads the project and is responsible for study integrity. GW, JS, ZH, KE, PM-A, KAS, RTE, CJ, PB and GH critically reviewed the study proposal and secured the research funding. JS, AS and KE lead collaboration sites. P-MA leads work-streams 2 and 4. RTE leads work-stream 3. GF manages the study and led the ethics submission. All authors contributed to the research protocol, provided edits and critiqued the manuscript for intellectual content.

ACKNOWLEDGMENTS

The authors are extremely grateful to Professor Anne Magriet Pot, Erasmus School of Policy and Health Management, Erasmus University, the Netherlands for her inspiration and encouragement to seek funding for this project, the Caban Group for their thoughtful and useful insights, and the research assistants (Danielle Proctor, Gwenllian Hughes, John Connaghan, Fatene Abakar Ismail) for piloting the case report forms and to Stéfanie Freel and Katrin Seeher of the World Health Organisation for their comments on the funding application and ongoing support for the project delivery.

FUNDING STATEMENT

This project is funded by the National Institute for Health Research (NIHR) Public Health Research programme (project reference NIHR130914). The views expressed are those of the authors and not necessarily those of the NIHR or the Department of Health and Social Care.

DATA AVAILABILITY STATEMENT

Data requests should be made with the Corresponding author.

COMPETING INTERESTS STATEMENT

The authors declare they have no competing interests.

For peer review only

REFERENCES

[1] World Health Organization. Global status report on the public health response to dementia [Internet]. Geneva: World Health Organization; 2021 Sep [cited 2022 Jan 28]. https://www.who.int/publications/i/item/9789240033245

[2] Wittenberg R, Hu B, Barraza-Araiza L, et al. Projections of older people with dementia and costs of dementia care in the United Kingdom, 2019–2040: Working paper [Internet]. London: Care Policy and Evaluation Centre; 2019 Nov [cited 2022 Jan 28]. https://www.alzheimers.org.uk/sites/default/files/2019-11/cpec_report_november_2019.pdf

[3] Lewis F, Karlsberg S, Sussex J, et al. Trajectory of dementia in the UK – making a difference: consulting report [Internet]. London: Office of Health Economics; 2014 Jun [cited 2022 Jan 28]. https://www.alzheimersresearchuk.org/wp-content/uploads/2015/01/OHE-report-Full.pdf

[4] Gilhooly KJ, Gilhooly ML, Sullivan MP, et al. A meta-review of stress, coping and interventions in dementia and dementia caregiving. *BMC Geriatr*. 2016 May 18;16:106. doi: 10.1186/s12877-016-0280-8

[5] Bremer P, Cabrera E, Leino-Kilpi H, et al. Informal dementia care: Consequences for caregivers' health and health care use in 8 European countries. *Health Policy*. 2015 Nov;119(11):1459-71. doi: 10.1016/j.healthpol.2015.09.014 [published Online First: 22 October 2022].

[6] Pinquart M, Sörensen S. Differences between caregivers and noncaregivers in psychological health and physical health: a meta-analysis. *Psychol Aging*. 2003 Jun;18(2):250-67. doi: 10.1037/0882-7974.18.2.250

For peer review only - http://bmjopen.bmj.com/site/about/guidelines.xhtml

[7] McCabe M, You E, Tatangelo G. Hearing Their Voice: A Systematic Review of Dementia Family Caregivers' Needs. *Gerontologist*. 2016 Oct;56(5):e70-88. doi: 10.1093/geront/gnw078 [published Online First: 21 April 2016].

[8] World Health Organization. Global action plan on the public health response to dementia 2017–2025 [Internet]. Geneva: World Health Organization; 2017 [cited 2022 Jan 28]. https://apps.who.int/iris/bitstream/handle/10665/259615/9789241513487-eng.pdf?sequence=1

[9] Dickinson C, Dow J, Gibson G, et al. Psychosocial intervention for carers of people with dementia: What components are most effective and when? A systematic review of systematic reviews. *Int Psychogeriatr*. 2017 Jan;29(1):31-43. doi: 10.1017/S1041610216001447 [published Online First: 26 September 2016].

[10] Masterson-Algar P, Allen MC, Hyde M, et al. Exploring the impact of Covid-19 on the care and quality of life of people with dementia and their carers: A scoping review. *Dementia*. Published Online First: 1 November 2021. https://doi.org/10.1177/14713012211053971

[11] Egan KJ, Pinto-Bruno ÁC, Bighelli I, et al. Online Training and Support Programs Designed to Improve Mental Health and Reduce Burden Among Caregivers of People With Dementia: A Systematic Review. *J Am Med Dir Assoc*. 2018 Mar;19(3):200-206.e1. doi: 10.1016/j.jamda.2017.10.023 [published Online First: 4 January 2018].

[12] Godwin KM, Mills WL, Anderson JA, et al. Technology-driven interventions for caregivers of persons with dementia: a systematic review. *Am J Alzheimers Dis Other Demen*. 2013 May;28(3):216-22. doi: 10.1177/1533317513481091 [published Online First: 25 March 2013].

For peer review only

[13] Gitlin LN, Marx K, Stanley IH, et al. Translating Evidence-Based Dementia Caregiving Interventions into Practice: State-of-the-Science and Next Steps. *Gerontologist*. 2015 Apr;55(2):210-26. doi: 10.1093/geront/gnu123 [published Online First: 17 February 2015].

[14] Peterson K, Hahn H, Lee AJ, et al. In the Information Age, do dementia caregivers get the information they need? Semi-structured interviews to determine informal caregivers' education needs, barriers, and preferences. *BMC Geriatr*. 2016 Sep;16(164). doi:10.1186/s12877-016-0338-7

[15] Christie HL, Martin JL, Connor J, et al. eHealth interventions to support caregivers of people with dementia may be proven effective, but are they implementation-ready? *Internet Interv*. 2019 Jul;18:100260. doi: 10.1016/j.invent.2019.100260

[16] Hopwood J, Walker N, McDonagh L, et al. Internet-Based Interventions Aimed at Supporting Family Caregivers of People With Dementia: Systematic Review. *J Med Internet Res*. 2018 Jun;20(6):e216. doi: 10.2196/jmir.9548

[17] Pinto-Bruno ÁC, Pot AM, Kleiboer A, et al. An Online Minimally Guided Intervention to Support Family and Other Unpaid Carers of People With Dementia: Protocol for a Randomized Controlled Trial. *JMIR Res Protoc*. 2019 Oct;8(10):e14106. doi: 10.2196/14106

[18] Mehta KM, Gallagher-Thompson D, Varghese M, et al. iSupport, an online training and support program for caregivers of people with dementia: study protocol for a randomized controlled trial in India. *Trials*. 2018 May;19(1):271. doi: 10.1186/s13063-018-2604-9

[19] Teles S, Ferreira A, Seeher K, et al. Online training and support program (iSupport) for informal dementia caregivers: protocol for an intervention study in Portugal. *BMC Geriatr*. 2020 Jan;20(1):10. doi: 10.1186/s12877-019-1364-z

For peer review only - http://bmjopen.bmj.com/site/about/guidelines.xhtml

[20] Baker JR, Jeon YH, Goodenough B, et al. What do children need to know about dementia? The perspectives of children and people with personal experience of dementia. *Int Psychogeriatr*. 2018 May;30(5):673-684. doi: 10.1017/S1041610217002022 [published Online First: 2 October 2017].

[21] National Institute for Health and Care Excellence (NICE). Methods for the development of NICE public health guidance [Internet]. 3rd ed. UK: NICE; c2006 [updated 2012 Sep; cited 2022 Jan 28]. https://www.nice.org.uk/process/pmg4/resources/methods-for-the-development-of-nice-public-health-guidance-third-edition-pdf-2007967445701

[22] Chan AW, Tetzlaff JM, Altman DG, et al. SPIRIT 2013 statement: defining standard protocol items for clinical trials. *Ann Intern Med*. 2013 Feb;158(3):200-7. doi: 10.7326/0003-4819-158-3-201302050-00583

[23] Join Dementia Research is funded by the Department of Health and delivered by the National Institute for Health Research in partnership with Alzheimer Scotland, Alzheimer's Research UK and Alzheimer's Society. www.joindementiaresearch.nihr.ac.uk [accessed 10/03/2021].

[24] Deeken F, Rezo A, Hinz M, et al. Evaluation of Technology-Based Interventions for Informal Caregivers of Patients With Dementia-A Meta-Analysis of Randomized Controlled Trials. *Am J Geriatr Psychiatry*. 2019 Apr;27(4):426-445. doi: 10.1016/j.jagp.2018.12.003. Epub 2018 Dec 6. PMID: 30642650.

[25] Sugimoto T, Sozu T, Hamasaki T. A convenient formula for sample size calculations in clinical trials with multiple co-primary continuous endpoints. *Pharm Stat*. 2012 Mar-Apr;11(2):118-28. doi: 10.1002/pst.505

For peer review only - http://bmjopen.bmj.com/site/about/guidelines.xhtml

[26] Sozu T, Sugimoto T, Hamasaki T, et al. Sample Size Determination in Clinical Trials with Multiple Endpoints. Springer International Publishing; 2015. https://doi.org/10.1007/978-3-319-22005-5

[27] Russell D, Hoare ZS, Whitaker R, et al. Generalized method for adaptive randomization in clinical trials. *Stat Med*. 2011 Apr;30(9):922-34. doi: 10.1002/sim.4175 [published Online First: 1 February 2011].

[28] Sörensen S, Pinquart M, Duberstein P. How effective are interventions with caregivers? An updated meta-analysis. *Gerontologist*. 2002 Jun;42(3):356-72. doi: 10.1093/geront/42.3.356

[29] Kitwood T. Dementia reconsidered: The person comes first. UK: Open University Press; 1997.

[30] Pot AM, Gallagher-Thompson D, Xiao LD, et al. iSupport: a WHO global online intervention for informal caregivers of people with dementia. *World Psychiatry*. 2019 Oct;18(3):365-366. doi: 10.1002/wps.20684

[31] Office for National Statistics (ONS). 2011 Census: Key statistics for Wales, March 2011 [Internet]. UK: ONS; 2012 Dec. Section 10. Proficiency in Welsh; [cited 2022 Jan 28]; https://www.ons.gov.uk/peoplepopulationandcommunity/populationandmigration/populationestimates/bulletins/2011censuskeystatisticsforwales/2012-12-11#proficiency-in-welsh

[32] Alzheimer's Society. Caring for a person with dementia: A practical guide. London: Alzheimer's Society, 2002 [reviewed 2019]. Available from: https://www.alzheimers.org.uk/get-support/publications-factsheets/caring-person-dementia-practical-guide

[33] Elsevier Ltd. MACRO (Version 4.9.0.8594) [Computer software]. London; c2018

For peer review only

[34] Vasileiou K, Barnett J, Thorpe S, et al. Characterising and justifying sample size sufficiency in interview-based studies: systematic analysis of qualitative health research over a 15-year period. *BMC Med Res Methodol*. 2018 Nov;18(1):148. doi: 10.1186/s12874-018-0594-7

[35] Ritchie J, Lewis J, Elam G. Designing and selecting samples. In: Ritchie J, Lewis J, editors. Qualitative research practice: a guide for social science students and researchers. London: SAGE Publications Ltd.; 2003. p.77–108.

[36] Moore GF, Audrey S, Barker M, et al. Process evaluation of complex interventions: Medical Research Council guidance. *BMJ*. 2015 Mar;350:h1258. doi: 10.1136/bmj.h1258

[37] O'Cathain A, Croot L, Duncan E, et al. Guidance on how to develop complex interventions to improve health and healthcare. *BMJ Open*. 2019 Aug;9(8):e029954. doi: 10.1136/bmjopen-2019-029954

[38] May C, Finch T, Mair F, et al. Understanding the implementation of complex interventions in health care: the normalization process model. *BMC Health Serv Res*. 2007 Sep;7:148. doi: 10.1186/1472-6963-7-148

[39] Brooke J. SUS: A 'quick and dirty' usability scale. In: Jordan PW, Thomas B, Weerdmeester BA, McClelland IL, editors. Usability Evaluation in Industry. London: Taylor Francis; 1996:189–94.

[40] Lancaster GA, Dodd S, Williamson PR. Design and analysis of pilot studies: recommendations for good practice. *J Eval Clin Pract*. 2004 May;10(2):307-12. doi: 10.1111/j..2002.384.doc.x

[41] Braun V, Clarke V. Using thematic analysis in psychology. *Qual Res Psychol*. 2006;3(2): 77-101. doi: 10.1191/1478088706qp063oa

For peer review only - http://bmjopen.bmj.com/site/about/guidelines.xhtml

[42] Pfadenhauer LM, Gerhardus A, Mozygemba K, et al. Making sense of complexity in context and implementation: the Context and Implementation of Complex Interventions (CICI) framework. *Implement Sci*. 2017 Feb;12(1):21. doi: 10.1186/s13012-017-0552-5

[43] Fenwick E, O'Brien BJ, Briggs A. Cost-effectiveness acceptability curves--facts, fallacies and frequently asked questions. *Health Econ*. 2004 May;13(5):405-15. doi: 10.1002/hec.903

[44] Wiltsey Stirman S, Baumann AA, Miller CJ. The FRAME: an expanded framework for reporting adaptations and modifications to evidence-based interventions. *Implement Sci*. 2019 Jun;14(1):58. doi: 10.1186/s13012-019-0898-y

[45] Dementia Engagement and Empowerment Project (DEEP). The DEEP-Ethics Gold Standards for Dementia Research [Internet]. Version 1. Exeter (UK): Innovations in Dementia; 2020 [cited 2022 Jan 28]. https://www.dementiavoices.org.uk/wp-content/uploads/2020/07/The-DEEP-Ethics-Gold-Standards-for-Dementia-Research.pdf

[46] Eysenbach G; CONSORT-EHEALTH Group. CONSORT-EHEALTH: improving and standardizing evaluation reports of Web-based and mobile health interventions. *J Med Internet Res*. 2011 Dec;13(4):e126. doi: 10.2196/jmir.1923

[47] Husereau D, Drummond M, Augustovski F, et al. Consolidated Health Economic Evaluation Reporting Standards 2022 (CHEERS 2022) Statement: Updated Reporting Guidance for Health Economic Evaluations. *Value Health*. 2022 Jan;25(1):3-9. doi: 10.1016/j.jval.2021.11.1351

[i] Bédard M, Molloy DW, Squire L, et al. The Zarit Burden Interview: a new short version and screening version. *Gerontologist*. 2001 Oct;41(5):652-7. doi: 10.1093/geront/41.5.652.

For peer review only - http://bmjopen.bmj.com/site/about/guidelines.xhtml

[ii] Andresen EM, Byers K, Friary J, et al. Performance of the 10-item Center for Epidemiologic Studies Depression scale for caregiving research. *SAGE Open Med*. 2013 Dec;1:2050312113514576. doi: 10.1177/2050312113514576.

[iii] Herdman M, Gudex C, Lloyd A, et al. Development and preliminary testing of the new five-level version of EQ-5D (EQ-5D-5L). *Qual Life Res*. 2011 Dec;20(10):1727-36. doi: 10.1007/s11136-011-9903-x [published Online First: 09 April 2011].

[iv] Wagnild G. The Resilience Scale User's Guide for the US English version of the Resilience Scale and the 14-item Resilience Scale (RS-14). Worden, MT: The Resilience Center; 2009.

[v] Spitzer RL, Kroenke K, Williams JB, et al. A brief measure for assessing generalized anxiety disorder: the GAD-7. *Arch Intern Med*. 2006 May;166(10):1092-7. doi: 10.1001/archinte.166.10.1092.

[vi] Annear MJ, Toye C, Elliott KJ, et al. Dementia knowledge assessment scale (DKAS): confirmatory factor analysis and comparative subscale scores among an international cohort. *BMC Geriatr*. 2017 Jul;17(1):168. doi: 10.1186/s12877-017-0552-y.

[vii] Hoefman RJ, Van Exel NJA, Brouwer WBF. iMTA Valuation of Informal Care Questionnaire (iVICQ). Version 1.0 (December 2011). Rotterdam: iBMG / iMTA, 2011.

[viii] Spruytte N, Van Audenhove C, Lammertyn F. The Scale for the Quality of the Current Relationship. Internal Report. Leuven: LUCAS-KULeuven; 2000.

[ix] Mulhern B, Rowen D, Brazier J, et al. Development of DEMQOL-U and DEMQOL-PROXY-U: generation of preference-based indices from DEMQOL and DEMQOL-PROXY

For peer review only

for use in economic evaluation. *Health Technol Assess*. 2013 Feb;17(5):v-xv, 1-140. doi: 10.3310/hta17050.

Figures

Figure 1. iSupport content.

Figure 2. Recruitment flowchart.

For peer review only

For peer review only - http://bmjopen.bmj.com/site/about/guidelines.xhtml

Figure 1: The content of iSupport for dementia carers

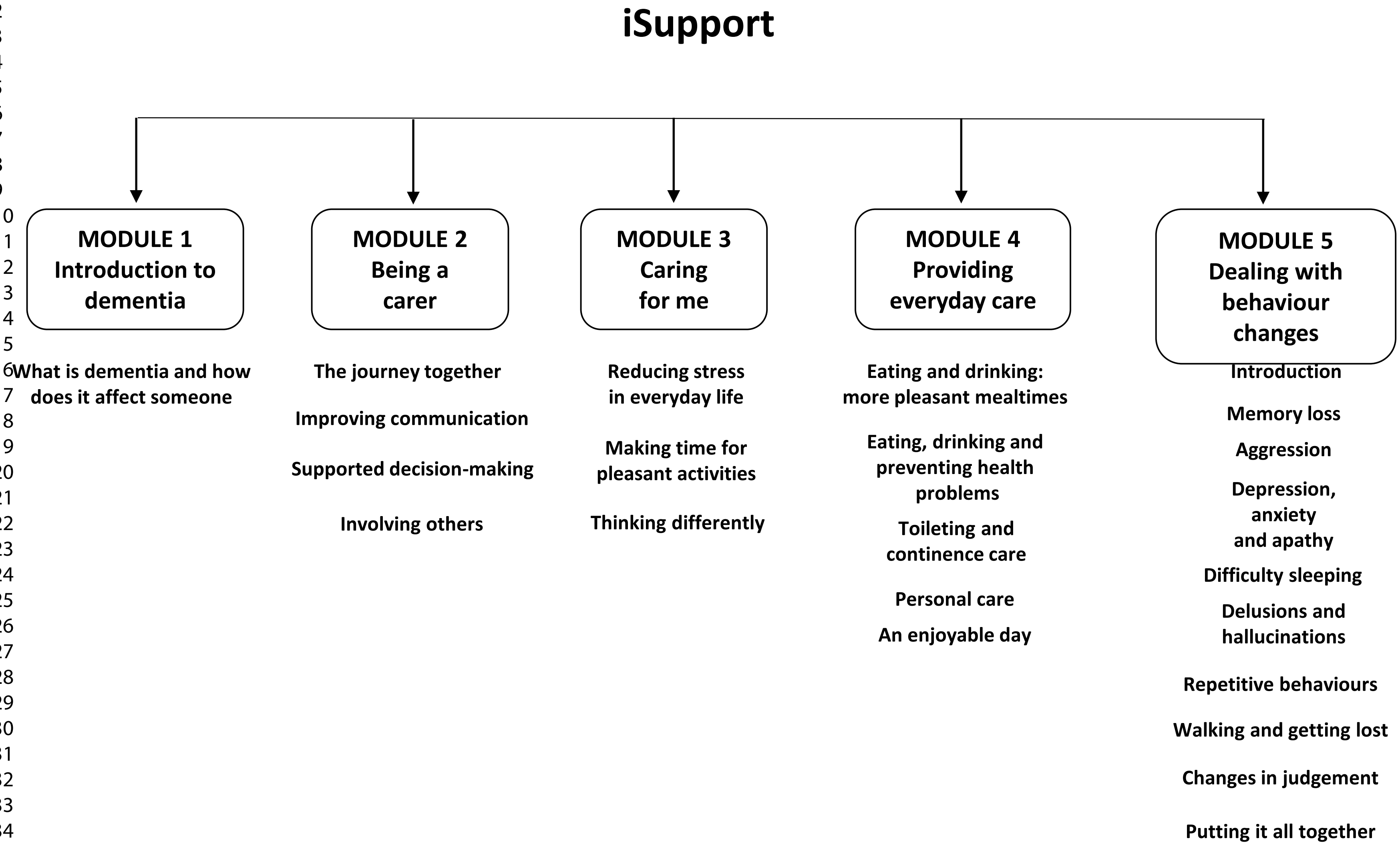

Figure 2: Recruitment flow chart

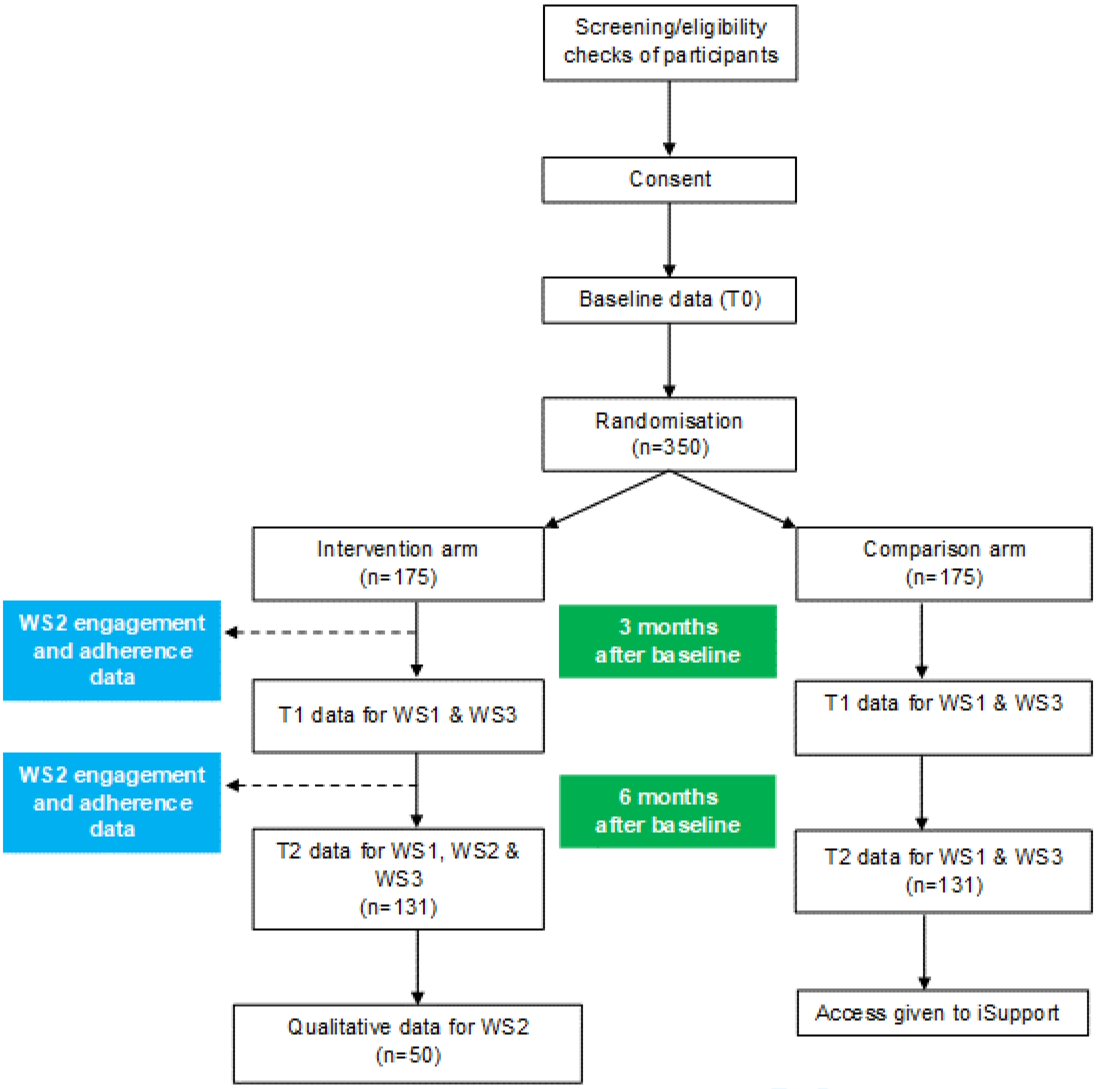

only

Supplementary Material

File 1 Objectives for each work-stream

WS1. A definitive pragmatic individually randomised controlled trial across Wales, Scotland and England, with a six-month nested internal pilot. This will:

• Determine progression of the definitive trial based on a go/review/stop criteria (nested internal pilot).
• Determine the effectiveness of 'iSupport' in reducing symptoms of distress and/or depression.

• Determine the effectiveness of 'iSupport' in reducing symptoms of anxiety.

• Determine the effectiveness of 'iSupport' in improving dementia knowledge, relationship quality and resilience.

• Describe the trial sample according to demographic/socioeconomic characteristics.

WS2. A process evaluation will be conducted in line with the established guidelines for process evaluations of complex evaluations[15,16] to determine the barriers and facilitators to the implementation of 'iSupport' at scale, and the extent it supports carers in the face of the ongoing or future COVID-19 pandemic. This will:
• Determine participant engagement and adherence to 'iSupport'.
• Explore the mechanisms of change.

• Identify the external factors to 'iSupport' which influence the delivery and function of the intervention.

• Explore the contextual factors that influence the scalability of 'iSupport' into wider contexts using the CICI framework.[17]

WS3. A parallel cost-effectiveness analysis, undertaken from both a public sector perspective (NHS, personal social services and local authorities), and a societal perspective (public sector plus opportunity costs). This will:
• Calculate the costs of implementing 'iSupport', including technical support and time spent supporting carers to use the tool.

• Explore patterns of, and estimate the cost of, health and social care resource use for carers in the 'iSupport' and comparison arms of the trial.

• Explore patterns of, and estimate the cost of, health and social care resource use for the care recipients of carers in the trial.

• Explore the opportunity cost of informal care through the measurement of informal care time, types of care task, impacts on carer's leisure and employment hours, and carers' willingness to pay for more support.

• Using QALYs derived from the EQ-5D-5L, determine the cost-effectiveness of 'iSupport' compared to the control condition; conduct secondary cost-effectiveness analyses using the Zarit Burden Interview[18] and the Centre for Epidemiological Studies of Depression Scale (CES-D10).[19,20]

For peer review only

File 2 Recruitment process

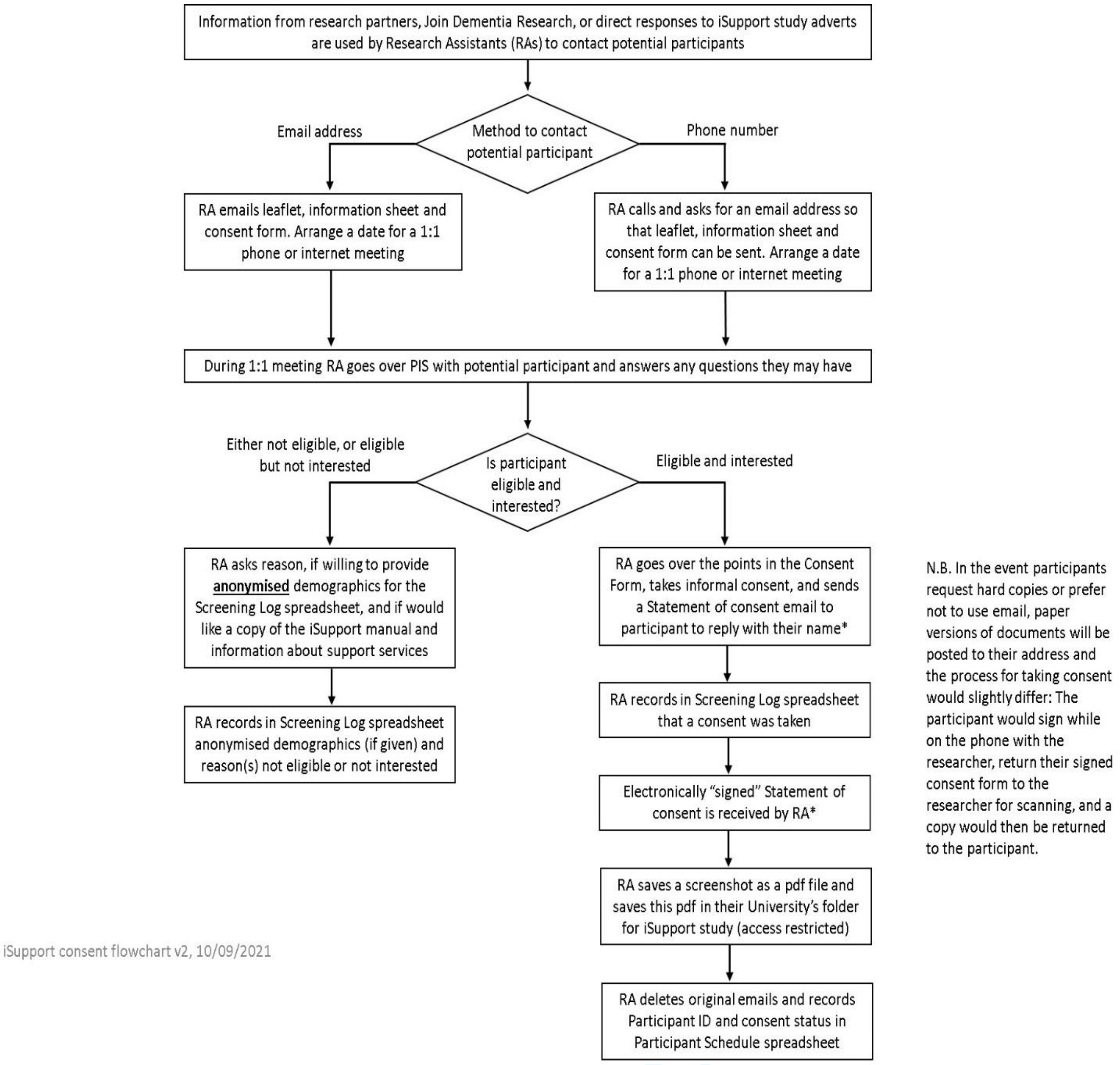

File 3 Feasibility study flowchart

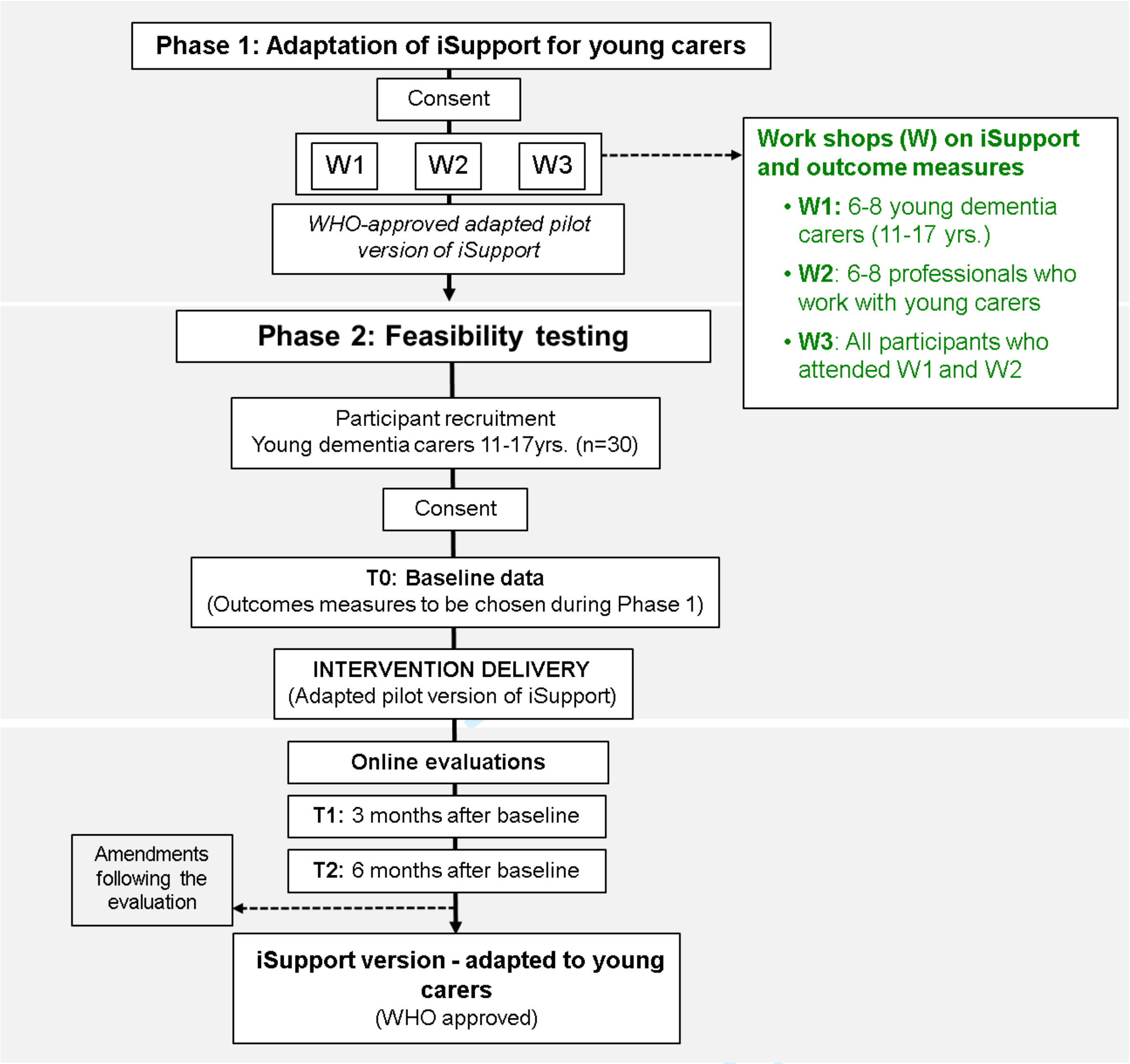

SPIRIT

STANDARD PROTOCOL ITEMS: RECOMMENDATIONS FOR INTERVENTIONAL TRIALS

SPIRIT 2013 Checklist: Recommended items to address in a clinical trial protocol and related documents*

	Section/item	Item No	Description
	**Administrative information**		
✓	Title	1	Descriptive title identifying the study design, population, interventions, and, if applicable, trial acronym
✓	Trial registration	2a	Trial identifier and registry name. If not yet registered, name of intended registry
✓		2b	All items from the World Health Organization Trial Registration Data Set
✓	Protocol version	3	Date and version identifier
✓	Funding	4	Sources and types of financial, material, and other support
✓	Roles and responsibilities	5a	Names, affiliations, and roles of protocol contributors
✓		5b	Name and contact information for the trial sponsor
✓		5c	Role of study sponsor and funders, if any, in study design; collection, management, analysis, and interpretation of data; writing of the report; and the decision to submit the report for publication, including whether they will have ultimate authority over any of these activities
✓		5d	Composition, roles, and responsibilities of the coordinating centre, steering committee, endpoint adjudication committee, data management team, and other individuals or groups overseeing the trial, if applicable (see Item 21a for data monitoring committee)
	**Introduction**		
✓	Background and rationale	6a	Description of research question and justification for undertaking the trial, including summary of relevant studies (published and unpublished) examining benefits and harms for each intervention
✓		6b	Explanation for choice of comparators
✓	Objectives	7	Specific objectives or hypotheses
✓	Trial design	8	Description of trial design including type of trial (eg, parallel group, crossover, factorial, single group), allocation ratio, and framework (eg, superiority, equivalence, noninferiority, exploratory)

For peer review only

Methods: Participants, interventions, and outcomes

✓	Study setting	9	Description of study settings (eg, community clinic, academic hospital) and list of countries where data will be collected. Reference to where list of study sites can be obtained
✓	Eligibility criteria	10	Inclusion and exclusion criteria for participants. If applicable, eligibility criteria for study centres and individuals who will perform the interventions (eg, surgeons, psychotherapists)
✓	Interventions	11a	Interventions for each group with sufficient detail to allow replication, including how and when they will be administered
✓		11b	Criteria for discontinuing or modifying allocated interventions for a given trial participant (eg, drug dose change in response to harms, participant request, or improving/worsening disease)
✓		11c	Strategies to improve adherence to intervention protocols, and any procedures for monitoring adherence (eg, drug tablet return, laboratory tests)
✓		11d	Relevant concomitant care and interventions that are permitted or prohibited during the trial
✓	Outcomes	12	Primary, secondary, and other outcomes, including the specific measurement variable (eg, systolic blood pressure), analysis metric (eg, change from baseline, final value, time to event), method of aggregation (eg, median, proportion), and time point for each outcome. Explanation of the clinical relevance of chosen efficacy and harm outcomes is strongly recommended
✓	Participant timeline	13	Time schedule of enrolment, interventions (including any run-ins and washouts), assessments, and visits for participants. A schematic diagram is highly recommended (see Figure)
✓	Sample size	14	Estimated number of participants needed to achieve study objectives and how it was determined, including clinical and statistical assumptions supporting any sample size calculations
✓	Recruitment	15	Strategies for achieving adequate participant enrolment to reach target sample size

Methods: Assignment of interventions (for controlled trials)

Allocation:

✓	Sequence generation	16a	Method of generating the allocation sequence (eg, computer-generated random numbers), and list of any factors for stratification. To reduce predictability of a random sequence, details of any planned restriction (eg, blocking) should be provided in a separate document that is unavailable to those who enrol participants or assign interventions

✓	Allocation concealment mechanism	16b	Mechanism of implementing the allocation sequence (eg, central telephone; sequentially numbered, opaque, sealed envelopes), describing any steps to conceal the sequence until interventions are assigned
✓	Implementation	16c	Who will generate the allocation sequence, who will enrol participants, and who will assign participants to interventions
✓	Blinding (masking)	17a	Who will be blinded after assignment to interventions (eg, trial participants, care providers, outcome assessors, data analysts), and how
✓		17b	If blinded, circumstances under which unblinding is permissible, and procedure for revealing a participant's allocated intervention during the trial
	**Methods: Data collection, management, and analysis**		
✓	Data collection methods	18a	Plans for assessment and collection of outcome, baseline, and other trial data, including any related processes to promote data quality (eg, duplicate measurements, training of assessors) and a description of study instruments (eg, questionnaires, laboratory tests) along with their reliability and validity, if known. Reference to where data collection forms can be found, if not in the protocol
✓		18b	Plans to promote participant retention and complete follow-up, including list of any outcome data to be collected for participants who discontinue or deviate from intervention protocols
✓	Data management	19	Plans for data entry, coding, security, and storage, including any related processes to promote data quality (eg, double data entry; range checks for data values). Reference to where details of data management procedures can be found, if not in the protocol
✓	Statistical methods	20a	Statistical methods for analysing primary and secondary outcomes. Reference to where other details of the statistical analysis plan can be found, if not in the protocol
✓		20b	Methods for any additional analyses (eg, subgroup and adjusted analyses)
✓		20c	Definition of analysis population relating to protocol non-adherence (eg, as randomised analysis), and any statistical methods to handle missing data (eg, multiple imputation)
	**Methods: Monitoring**		
✓	Data monitoring	21a	Composition of data monitoring committee (DMC); summary of its role and reporting structure; statement of whether it is independent from the sponsor and competing interests; and reference to where further details about its charter can be found, if not in the protocol. Alternatively, an explanation of why a DMC is not needed

✓		21b	Description of any interim analyses and stopping guidelines, including who will have access to these interim results and make the final decision to terminate the trial
✓	Harms	22	Plans for collecting, assessing, reporting, and managing solicited and spontaneously reported adverse events and other unintended effects of trial interventions or trial conduct
✓	Auditing	23	Frequency and procedures for auditing trial conduct, if any, and whether the process will be independent from investigators and the sponsor
	**Ethics and dissemination**		
✓	Research ethics approval	24	Plans for seeking research ethics committee/institutional review board (REC/IRB) approval
✓	Protocol amendments	25	Plans for communicating important protocol modifications (eg, changes to eligibility criteria, outcomes, analyses) to relevant parties (eg, investigators, REC/IRBs, trial participants, trial registries, journals, regulators)
✓	Consent or assent	26a	Who will obtain informed consent or assent from potential trial participants or authorised surrogates, and how (see Item 32)
N/A		26b	Additional consent provisions for collection and use of participant data and biological specimens in ancillary studies, if applicable
✓	Confidentiality	27	How personal information about potential and enrolled participants will be collected, shared, and maintained in order to protect confidentiality before, during, and after the trial
✓	Declaration of interests	28	Financial and other competing interests for principal investigators for the overall trial and each study site
✓	Access to data	29	Statement of who will have access to the final trial dataset, and disclosure of contractual agreements that limit such access for investigators
N/A	Ancillary and post-trial care	30	Provisions, if any, for ancillary and post-trial care, and for compensation to those who suffer harm from trial participation
✓	Dissemination policy	31a	Plans for investigators and sponsor to communicate trial results to participants, healthcare professionals, the public, and other relevant groups (eg, via publication, reporting in results databases, or other data sharing arrangements), including any publication restrictions
✓		31b	Authorship eligibility guidelines and any intended use of professional writers
✓		31c	Plans, if any, for granting public access to the full protocol, participant-level dataset, and statistical code

For peer review only - http://bmjopen.bmj.com/site/about/guidelines.xhtml

Appendices

N/A	Informed consent materials	32	Model consent form and other related documentation given to participants and authorised surrogates
N/A	Biological specimens	33	Plans for collection, laboratory evaluation, and storage of biological specimens for genetic or molecular analysis in the current trial and for future use in ancillary studies, if applicable

*It is strongly recommended that this checklist be read in conjunction with the SPIRIT 2013 Explanation & Elaboration for important clarification on the items. Amendments to the protocol should be tracked and dated. The SPIRIT checklist is copyrighted by the SPIRIT Group under the Creative Commons "Attribution-NonCommercial-NoDerivs 3.0 Unported" license.

For peer review only

BMJ Open

The effects of an e-health intervention ('iSupport') for reducing distress of dementia carers: protocol for a randomised controlled trial and feasibility study

Journal:	*BMJ Open*
Manuscript ID	bmjopen-2022-064314.R1
Article Type:	Protocol
Date Submitted by the Author:	28-Jul-2022
Complete List of Authors:	Windle, Gill; Bangor University<br>Flynn, Greg; Bangor University<br>Hoare, Zoe; Bangor University, North Wales Organisation for Randomised Trials in Health<br>Masterson-Algar, Patricia; Bangor University College of Health and Behavioural Sciences, School of Health Sciences<br>Egan, Kieren; University of Strathclyde, Department of Computer and Information Science<br>Edwards, Rhiannon; Bangor University, Centre for Health Economics & Medicines Evaluation<br>Jones, Carys; Bangor Univ<br>Spector, Aimee; University College London (UCL), Department of Clinical, Educational and Health Psychology<br>Algar-Skaife, Katherine; NTNU), Department of Neuro-medicine and Movement Science<br>Hughes, Gwenllian; Bangor University<br>Brocklehurst, P; Bangor University<br>Goulden, Nia; Bangor University, NWORTH CTU<br>Skelhorn, Debbie; Bangor University<br>Stott, Joshua; University College London Research Department of Clinical Educational and Health Psychology
**Primary Subject Heading**:	Mental health
Secondary Subject Heading:	Mental health, Health informatics
Keywords:	Dementia < NEUROLOGY, Telemedicine < BIOTECHNOLOGY & BIOINFORMATICS, HEALTH ECONOMICS

SCHOLARONE™ Manuscripts

For peer review only - http://bmjopen.bmj.com/site/about/guidelines.xhtml

The effects of an e-health intervention 'iSupport' for reducing distress of dementia carers: protocol for a randomised controlled trial and feasibility study

Authors

Gill Windle[1], Greg Flynn[1], Zoë Hoare[1], Patricia Masterson-Algar[1], Kieren Egan[2], Rhiannon Tudor Edwards[1], Carys Jones[1], Aimee Spector[3], Katherine Algar-Skaife[4], Gwenllian Hughes[1], Paul Brocklehurst[1], Nia Goulden[1], Debbie Skelhorn[1], Joshua Stott[3].

[1] School of Medical and Health Sciences, Bangor University, Bangor, UK

[2] Department of Computer and Information Science, University of Strathclyde, UK

[3] Department of Clinical, Educational and Health Psychology, University College London (UCL), London, UK

[4] Department of Neuro-medicine and Movement Science (INB), Norwegian University of Science and Technology (NTNU), Trondheim, Norway.

Corresponding author: Professor Gill Windle, DSDC Wales Research Centre/Ageing and Dementia @ Bangor, School of Medical and Health Sciences, Bangor University, Fron Heulog, Bangor, Gwynedd, Wales. g.windle@bangor.ac.uk

Word count for article summary and main text =3588 (excluding title page, abstract, references, figures and tables).

For peer review only

ABSTRACT

Introduction: In the United Kingdom, National Health Service (NHS) guidelines recommend that informal carers of people living with dementia should be offered training to help them develop care skills and manage their own physical and mental health. The World Health Organization (WHO) recommend access to affordable, proven, well-designed, online technologies for education, skills-training and support for dementia carers. In response to these recommendations, this multi-site randomised controlled trial (RCT) is the first study in the UK to evaluate the clinical and cost-effectiveness of an online support programme developed by the WHO called 'iSupport for dementia carers.'

Methods and analysis: 350 informal carers (age 18+) living in Britain who self-identify as experiencing stress and depression will be recruited. They will be randomised to receive 'iSupport', or standardised information about caring for someone with dementia (control-comparison). Data will be collected via videoconferencing (e.g. Zoom) or telephone interview at baseline, three months and six months. Intention-to-treat analysis will ascertain effectiveness in the primary outcomes (distress and depression) and combined cost and quality adjusted life year (QALY) data will be used to assess cost-effectiveness compared with usual care from a public sector and wider societal perspective. A mixed-methods process evaluation with a sub-group of carers in the intervention (~N=50) will explore the barriers and facilitators to implementing 'iSupport'. A non-randomised feasibility study will adapt 'iSupport' for young carers (N=38 participants, age 11-17).

For peer review only - http://bmjopen.bmj.com/site/about/guidelines.xhtml

Ethics and dissemination: The research plan was scrutinised by National Institute for Health Research reviewers ahead of funding being awarded. Ethical approval was granted by Bangor University's School of Health and Medical Sciences Academic Ethics Committee, reference number 2021-16915. Dissemination plans include delivering events for stakeholders, social media, a project website, developing policy briefings, presenting at conferences and producing articles for open access publications.

Registration details: Trial registration number ISRCTN17420703.

Keywords: dementia; carers; iSupport; technology; internet; website; randomized controlled trial; protocol; process evaluation; cost effectiveness; young carers.

For peer review only

ARTICLE SUMMARY

Strengths and limitations of this study

- iSupport for dementia carers was developed by experts at the WHO and is based on techniques with proven therapeutic efficacy, consequently the content is informed by a considerable body of evidence.
- The 'real world' application of the randomized controlled trial requires carers to self-identify as experiencing some level of stress or depression, but some may have mild symptoms, limiting the potential for improving these primary outcomes.
- Although the research assistants will be 'blind' to the randomisation, a limitation of the study includes being unable to completely blind the participants to their respective allocation (iSupport or information about being a carer).
- Remote data collection and intervention delivery will potentially reach a broader and more diverse range of carers beyond the geographical boundaries often experienced through in-person data collection, however this could also create challenges for recruiting to target.
- The feasibility study will work with young people to generate valuable information leading to an adapted version of 'iSupport' for young carers.

For peer review only - http://bmjopen.bmj.com/site/about/guidelines.xhtml

INTRODUCTION

'Dementia' is an umbrella term for a cluster of symptoms that characterise neurodegenerative changes, decline and loss of cognitive functioning. Dementia is one of the leading causes of care dependency, disability and death around the world.[1] The number of people living with dementia is predicted to increase globally, and it is estimated the number of people living with dementia in the UK will increase 80% by 2040.[2] The limited medical treatments available for people living with dementia mean that in the UK, most people living with dementia are cared for at home,[3] supported by a family member or friend who often performs care tasks similar to those carried out by paid health or social service providers. The detrimental impact of caregiving on the physical and mental health of informal carers is well-documented;[4,5] a meta-analysis found carers were more stressed, depressed, and had lower levels of subjective well-being, physical health, and self-efficacy than non-carers.[6]

Dementia carers have expressed a need for: a) relevant information and knowledge; b) support with the management of care recipients' functioning, behavioural and psychological symptoms; c) support with their own physical and mental health; d) support regarding their unbalanced social life.[7] In the face of these significant challenges, Action Area 5 of the World Health Organization's Global Action Plan on Dementia 2017-2025 prioritises supporting carers, calling for the provision of accessible evidence-based information to improve knowledge and skills, and prevent stress and health problems.[8]

To address these challenges the World Health Organization (WHO) developed 'iSupport', an evidence-informed e-health intervention designed to help dementia carers provide good care and take care of themselves. The content reflects evidence that the most effective interventions for carers' psychological health should incorporate both an educational

For peer review only - http://bmjopen.bmj.com/site/about/guidelines.xhtml

component to enhance knowledge, and a therapeutic component, such as Cognitive Behavioural Therapy /cognitive reframing.[9] Such interventions are often delivered in-person, however the ongoing Covid-19 pandemic led to reductions, delays and withdrawal of many support services for carers.[10] Online interventions could be one solution to providing support, negating general accessibility barriers such as carers' time constraints or needing to travel to receive care and support,[11] due to their convenience of use, low delivery costs, and the ability to negate geographical barriers.[12] The potential for scalability is also relevant, as few e-health interventions for carers are implemented outside a research setting.[13,14,15] However, despite their potential, the evidence base remains limited and high-quality studies are required to enable definitive conclusions about their effectiveness.[16] In response, this study aims to contribute to this growing area of healthcare delivery.

'iSupport' is in the process of global implementation and there is research underway in The Netherlands, India and Portugal,[17,18,19] but to date, there is no published evidence as to the effectiveness of 'iSupport'. This will be the first study to examine the effectiveness and cost effectiveness of a globally targeted e-health intervention in a majority English-speaking population of dementia carers. It will also evaluate the feasibility of adapting 'iSupport' for young carers (ages 11-17). It is vital that current and future carers have access to education programmes that are tailored to address their particular needs,[20] as current generic dementia support services are not able to address the specific challenges young carers face.

Research questions

1. Are carer distress and depression (primary outcomes) significantly reduced in participants allocated to receive 'iSupport' compared to participants allocated to a control-comparison group receiving standardised information about caring for someone with dementia?

For peer review only - http://bmjopen.bmj.com/site/about/guidelines.xhtml

2. Are symptoms of anxiety (secondary outcome) significantly reduced and resilience, relationship quality and dementia knowledge (secondary outcomes) significantly increased in participants allocated to receive 'iSupport' compared to participants allocated to the control-comparison group?

3. What are participant and contextual barriers and facilitators to implementation of 'iSupport'?

4. What potential mechanisms might underpin changes in outcomes from using 'iSupport'?

5. What is the cost-effectiveness of 'iSupport' compared to standardised information about dementia?

6. Is it feasible and acceptable to digitally deliver a refined 'iSupport' to young carers?

7. What are the carers' perspectives of 'iSupport' in relation to supporting them in an ongoing or future repeated pandemic such as COVID-19?

METHODS AND ANALYSIS

Study design

'iSupport' for dementia carers is a multi-centre randomised controlled trial (RCT) and feasibility study composed of four workstreams (WS). WS1 will evaluate the effectiveness of 'iSupport' (compared to a control-comparison) in reducing carer distress and symptoms of depression (multiple primary outcomes), reductions in anxiety, improvements in resilience, relationship quality, and dementia knowledge (secondary outcomes). WS2 (process evaluation) will examine how participants engaged with 'iSupport', whether there are any barriers to its uptake, and any perceived benefits for the carer. WS3 (health economic evaluation) will calculate the cost-effectiveness of 'iSupport' from a public sector perspective[21] and from a wider societal perspective. WS4 (feasibility study) will adapt 'iSupport' for young carers, and assesses the feasibility, acceptability and uptake of conducting a larger trial. Supplementary File 1 contains the objectives for each workstream. This protocol was developed according to the SPIRIT (2013) checklist[22]. The study runs for

For peer review only - http://bmjopen.bmj.com/site/about/guidelines.xhtml

36 months (1st January 2021 to 31st December 2023). At the end of their involvement in the study, all participants will receive information about regional support services and a £20 voucher.

RCT participant recruitment

Carers living in England, Scotland and Wales will be recruited between December 2021 and January 2023 by researchers working from Bangor University (co-ordinating centre), University College London, or University of Strathclyde (collaborating sites). Researchers will advertise the study through social media; our study partners (Alzheimer Scotland and Carers Trust Wales) and other non-statutory organisations will advertise the study to regional groups through their networks; and the Join Dementia Research (JDR)[23] register will be used to identify potential participants (Supplementary File 2). All carers who express an interest in taking part will be sent a consent form, information sheet and be invited to discuss their involvement with a researcher in a one-to-one videoconferencing or phone meeting, when the researcher would also assess their eligibility (Table 1).

Table 1: Eligibility criteria for the RCT.

Inclusion criteria	1) Adults (age 18+) who self-identify as an unpaid carer of a person with dementia who is not living in a full-time care facility, caring at least weekly for at least 6 months.<br>2) Self-identify as experiencing at least some stress, depression or anxiety.<br>3) The care recipient has to have a confirmed diagnosis of dementia through self-report of the carer.
Exclusion criteria	1) Receiving psychological treatment from a mental health specialist at the time of recruitment.

For peer review only - http://bmjopen.bmj.com/site/about/guidelines.xhtml

2) Unable to comprehend written English.

3) No access to the internet.

4) Unable to give informed consent to the trial.

5) Have previously used 'iSupport' materials in the last 12 months.

A nested internal pilot study at each site will monitor progression criteria over the first six months of recruitment. Go/review/stop criterion will be assessed by the study's independent data monitoring committee (IDMC), and decisions about the study conduct will be made in consultation with the trial steering committee (TSC) and the Trial Management Group (TMG).

RCT sample size and randomisation

A meta-analysis reported that technology-based interventions for informal carers of people living with dementia are effective in reducing both depression and burden outcomes.[24] Consequently both are important outcomes for carers, and the sample size considers these as multiple primary endpoints at six months. The multiple primary endpoint estimator in the R package [25,26] with power of 90% and significance set to 2.5%, established that 262 participants are required at six months to have the potential to detect an effect in at least one of these outcomes. The attrition rate was based on 9 dementia intervention studies, where the mean retention rate was 15.33% (range 2%-24%). Accommodating a 25% attrition rate by six months, the RCT will recruit and randomise 350 participants. Randomisation uses dynamic allocation to protect against subversion.[27] This ensures the trial maintains good balance to the allocation ratio of 1:1, both within each stratification variable and overall for the trial. Stratification variables will be site, along with age and gender, previously found to influence the outcome measure of caregiver distress.[28]

For peer review only - http://bmjopen.bmj.com/site/about/guidelines.xhtml

RCT 'iSupport' intervention

iSupport is an internet-based psychoeducation and skills development intervention that can be accessed through a personal computer, tablet or mobile phone. The theoretical underpinnings of 'iSupport' are based on person-centred care, recognising that dementia care should reflect the individual's needs, personality and abilities[29] and are integrated into the interactive content of 'iSupport'. The self-care techniques are based on theoretically informed programmes with some evidence for benefits, including psychoeducation, relaxation, behavioural activation, cognitive reframing, and problem-solving.[30] Participants will access iSupport in their own homes or a place where they are able to access the internet.

'iSupport' consists of five main themes and twenty-three accompanying exercises (Figure 1). Each exercise takes approximately 5-15 minutes and follows the same format: information about a topic presented; short interactive exercises and questions with instant feedback on responses; a summary of the lesson; a relaxation exercise. 'iSupport' is based on personal choice: carers can construct their own personalised plan and access which sessions they feel are most relevant to them at that point in time. Participants will be advised to use 'iSupport' regularly in order to obtain the most benefit. They will be provided with the contact details of an 'e-coach' (member of the research team), who will explain anything that is not clear about the 'iSupport' programme. The 'e-coach' will contact participants allocated to intervention shortly after randomisation, 1 month later and 2 months later (if required by the participant). 'iSupport' will be translated into Welsh following WHO adaptation guidelines. Approximately one-fifth of the Welsh population speak Welsh[31] and the Welsh Government is committed to offering bilingual services as part of health care provision. To improve access, an audio/read aloud function is included in the iSupport programme.

(Figure 1)

For peer review only - http://bmjopen.bmj.com/site/about/guidelines.xhtml

RCT control-comparison to iSupport

The control-comparison group will receive an information booklet (online and/or hard copy) about caring for someone with dementia, developed by the Alzheimer's Society.[32] Alongside this education, carers will receive care-as-usual. They can search for other information or seek help from other providers. Participants allocated to the control-comparison group will be provided with access to 'iSupport' at the end of data collection**.**

RCT data collection

Case report forms (CRFs) were initially piloted by researchers, and adjustments made to reduce the time-burden to participants without affecting the study's ability to address the research questions. Data will be collected at three time-points: Baseline (T0), 3-months post-baseline (T1 follow-up), and 6-months post-baseline (T2 follow-up). Researchers will interview participants by videoconferencing or phone. Following the baseline interview, researchers will perform the randomisation and the CI or trial manager will email the participant their group allocation details. Follow-up interviews will be administered by researchers who are blinded to group allocation. An acceptable tolerance for follow-ups will be up to 2 weeks earlier and up to 4 weeks later than the exact T1 or T2 date. Figure 2 shows the flow of the participants through the study.

(Figure 2)

All data will be entered into an electronic database (MACRO),[33] and the study statistician will periodically monitor data quality. Table 2 shows the outcome measures, order of administration and the relevance for each workstream.

For peer review only

Table 2: Data collection for iSupport RCT

Questionnaire or study-specific questions	Time point	Workstream
Local COVID-19 alert level at date of assessment	T0, T1, T2	1,2,3
Demographic questions	T0	1,2,3
Employment, marital status, and living situation questions	T0, T1, T2	1,2,3
12-item Zarit Burden Interview (ZBI-12)*[i]	T0, T1, T2	1,3
10-item Centre for Epidemiological Studies Depression Scale (CES-D-10)*[ii]	T0, T1, T2	1,3
EQ-5D-5L[iii]	T0, T1, T2	3
Resilience Scale-14 (RS-14)[iv]	T0, T1, T2	1
Generalised Anxiety Disorder Questionnaire (GAD-7)[v]	T0, T1, T2	1
Dementia Knowledge Assessment Scale (DKAS)[vi]	T0, T1, T2	1
Adapted Erasumus iMTA informal care questionnaire[vii]	T0, T1, T2	3
Service use questions	T0, T1, T2	3
Quality of the Carer-Patient Relationship (QCPR)[viii]	T0, T1, T2	1
Dementia Quality of Life - Proxy measure (DEMQOL-Proxy)[ix]	T0, T1, T2	1,3
Researcher remains blinded to allocation question	T1, T2	1

*indicates primary outcome measure for WS1

WS2 Process evaluation sampling and data collection

The process evaluation utilises three different approaches to data collection:

1. Semi-structured interviews will be undertaken with up to 50 of carers in the intervention group following their T2 interview. The choice of sample size in qualitative research is an area of debate,[34] however our decision was informed by Ritchie and colleagues[35] who recommend that studies employing individual interviews should undertake no more than 50

For peer review only - http://bmjopen.bmj.com/site/about/guidelines.xhtml

interviews in order to manage the complexity of the analysis. Baseline data will inform a purposive sampling strategy and a qualitative sampling matrix will be developed. This matrix will include a diverse range of participant demographic characteristics such as age, gender and caring responsibilities and differences in scores across the the ZBI-12 and the CES-D-10 scores (low, medium, high).

The interview topics will be guided by the process evaluation parameters described in recognised frameworks,[36,37] and drawing upon theoretical models such as Normalisation Process Theory (NPT).[38] Motives for declining participation will also be noted where consent is given, to understand any barriers to participation and potential selection bias.

2. Data from the online platform will be collected regarding usability (e.g. frequency and length of use, which modules/sessions/pages users most frequently visit, average time spend on each module/session/page, whether accessed from tablet, PC or mobile phone). The number of contacts with the e-coach will be recorded.

3. An online evaluation questionnaire will collect quantitative data from all study participants in the intervention arm and will be administered at 6-month follow-up (T2).[39] This questionnaire will evaluate the overall usability and acceptability of the 'iSupport' platform in conjunction with all other data collection methods.

WS4 Feasibility study: participant recruitment

Young carers and professionals who have regular contact with young carers will be recruited through stakeholders' networks, social media, and national carers associations (Table 3). Researchers will approach parents or legal guardians of participants under the age of 16 to

For peer review only - http://bmjopen.bmj.com/site/about/guidelines.xhtml

explain their child's involvement and obtain their consent from them. Supplementary File 3 visualises the phases of the feasibility study.

Table 3: Feasibility study eligibility criteria

	**Young carers**	**Professionals**
Inclusion criteria	1) Young people between the ages of 11 - 17 who self-identify as a carer of a person with dementia who is not living in a full-time care facility, caring at least weekly for at least 6 months.<br>2) The care recipient has to have a confirmed diagnosis of dementia (through self-report of the carer).	1) Have regular contact with young people and young carers (e.g. teaching staff involved in pastoral care, young carer charity workers, social workers in children's services.
Exclusion criteria	1) Receiving treatment from Child and Adolescent Mental Health Services (CAMHS) at the time of recruitment.<br>2) Unable to comprehend written English.<br>3) No access to the internet.<br>4) Have previously used 'iSupport' materials in the last 12 months.	1) No regular contact with young people and young carers as part of their work.<br>2) Unable to comprehend written English.<br>3) No access to the internet.

For peer review only - http://bmjopen.bmj.com/site/about/guidelines.xhtml

WS4: data collection

Phase 1: Adapting 'iSupport' for young carers

Three x 3-hour workshops will be conducted either in person or using videoconferencing software (e.g. Zoom, Teams or Skype) depending on the government guidelines regarding COVID-19 and safety. At least two weeks before the workshops, participants will be given online access to 'iSupport' and printed materials for annotations. Workshop One will recruit 6-8 young carers to discuss their care-giving experiences, which aspects are reflected or missing in 'iSupport', and opinions on the content and style of the intervention. Workshop Two will undertake a similar exercise with 6-8 professionals who work with young carers. Feedback will be used to refine 'iSupport', which will be shared in Workshop Three with all participants who attended the first two workshops in order to produce a "final" version. Discussions around which outcomes are most important for young carers in relation to 'iSupport' will be used to adapt the CRF from the RCT for Phase 2.

Phase 2: Feasibility testing 'iSupport' for younger dementia carers

Young carers will test the feasibility of using the refined 'iSupport' and following the RCT procedures (except randomisation will not be required). After T2 data collection, participants will complete an online evaluation of their experience using 'iSupport'. Informed by a methodological framework,[40] a sample of n=30 for phase 2 will provide enough information on the acceptability of the intervention, the appropriateness of data collection forms, the feasibility of recruitment and consent procedures, and the most appropriate primary outcome measures.

For peer review only - http://bmjopen.bmj.com/site/about/guidelines.xhtml

Data analysis plans

WS1 (research questions 1 and 2).

WS1 Primary analysis is an intention-to-treat (ITT) analysis, blinded to treatment allocation. The primary assessment for effectiveness will be adjusted estimates of the ZBI-12 and CES-D-10 scores between the two groups assessed at 6 months. A linear mixed-effects model adjusting for baseline scores, randomising site (random effect), and stratification variables will be fitted for each of the two primary outcomes. Similar models will be fitted for all continuous secondary outcomes. All estimates of effect will be presented together with 95% confidence intervals. The aim is to minimse missing data; however, predictors of missingness will be investigated using regression models and any predictors found will be considered for inclusion in the models. Multiple imputation will address missing scores where appropriate. Complier Average Causal Effect (CACE) analysis will assess the impact of the number of times the 'iSupport' intervention was accessed. A sensitivity analysis will assess any impact of the outcome measures being completed in Welsh. A full statistical analysis plan will be written and agreed with the independent committees before completion of the data collection.

WS2 Process evaluation (research questions 3, 4 and 7).

Qualitative interview data analysis will be professionally transcribed verbatim and thematically analysed[41] using NVivo. Results will also be applied to aspects of the Context and Implementation of Complex Interventions (CICI) checklist[42], which may reflect implementation in a 'real world' setting. This analysis will reveal the experiences of using iSupport and its delivery, the barriers and facilitators to its uptake and continued use, and the perceived benefits for the carer participating in iSupport and the person they are caring for. Descriptive analyses will profile the System Usability Scale and intervention platform

For peer review only - http://bmjopen.bmj.com/site/about/guidelines.xhtml

metrics regarding usability (e.g. most/least frequently visited pages, the most ‘popular’ modules/sessions).

WS3 Health economic evaluation (research question 5).

Primary analysis will be an ITT analysis as per WS1. Cost and quality-adjusted life years data will be combined to calculate an incremental cost-effectiveness ratio. Cost-effectiveness acceptability curves[43] will show the probability that ‘iSupport’ is cost-effective compared to the control-comparison for a range of willingness-to-pay thresholds. Secondary cost-effectiveness analyses will calculate the cost per unit change in the primary outcome measures. A sub-group analysis will be conducted on the number of times that carers in the intervention group accessed ‘iSupport’. Deterministic sensitivity analyses will be conducted to vary the costs of inputs.

WS4 feasibility study (research question 6).

Data from Phase 1 workshops will be selectively transcribed, analysed and reported according to established guidance[44]. All quantitative data collected during Phase 2 will be presented descriptively. No inferential testing will be undertaken for this data. The mean change from baseline, associated variances, and 95% confidence intervals will be calculated for all selected outcomes. Consideration will be given to the applicability of these outcomes for development into a protocol for a future RCT if the acceptability of the intervention is proven. Success will be defined as acceptability of the recruitment and consent procedure, data collection tools, intervention content and delivery to participants, as well as compliance.

Patient and public involvement

We involved people living with dementia and their carers in the development of this research. This was achieved by collaborating with the ‘Caban group of dementia educators’,

For peer review only - http://bmjopen.bmj.com/site/about/guidelines.xhtml

established and supported by the lead applicant's research centre. The group raised a number of points for the team to consider, with ‘fear of using the internet’ being one area of concern. The group felt a person should be available to help people with iSupport. In response we built in provision for an ‘e-coach’ to support participants randomised to receive iSupport. Co-applicant Hughes is a young adult carer for her father living with Vascular Dementia and felt the needs of young carers are often overlooked and neglected. She has contributed to the development of this research, especially the conceptualisation of the study design and suggestions for the delivery of WS4, and is assisting with this phase. We will meet with the CABAN group on a regular basis over the study duration, and at a previous meeting we discussed how a visual participant information sheet could aid recruitment in line with dementia research standards,[45] and that using videoconferencing software would be preferrable to phone calls for arranging and conducting remote interviews. Feedback from this meeting was further referred to when drafting other study materials for consistency.

ETHICS AND DISSEMINATION

iSupport was granted ethical approval by Bangor University’s School of Medical and Health Sciences Academic Ethics Committee (AEC), reference number 2021-16915. All researchers are fully trained in the study procedures and receive regular supervision. A data management and monitoring plan ensure adherence to the principles of Good Clinical Practice and relevant regulations over the course of the study, and to effectively audit the day-to-day conduct at each site. Carers will be provided with clear information and given time to ask questions and consider whether to participate before providing consent (Supplementary File 4). Through the content of our information sheets and consent forms, as well as contact with the research team, participants will understand that they can refuse to participate or withdraw at any time.

For peer review only - http://bmjopen.bmj.com/site/about/guidelines.xhtml

Changes to the study protocol will be agreed by the funder and an ethics amendment submitted to the AEC.

Our research products will include peer-reviewed academic papers, Plain English/Cymraeg Clir summaries of findings, articles for practitioner magazines and a project website. All academic outputs will conform to the reporting procedures in the relevant methodology guidelines (e.g. CONSORT e-health[46]). Economic evaluation findings will be reported according to the recently updated CHEERS checklist, highlighting the role of PPIE relating to health economics.[47] We will present at conferences, conduct public and stakeholder events, and produce policy briefings.

Our research activities will generate new versions of the iSupport platform for Welsh-language speakers, young carers, and a UK-focused version with audio function. If our research shows iSupport is effective, health and care providers, pastoral care teams in schools, and charitable organisations will be able to recommend an evidence-based online support service to dementia carers that will be publicly available for use at no cost. We hope this will improve policy and practice around delivering support to dementia carers. For example UK health and social care could recommend the adapted versions of iSupport in their dementia guidelines. This could reduce demand on community teams at post/diagnosis and initial stages of dementia.

Forthcoming in 2022 in a related project, we will be working in partnership with community organisations to translate and adapt iSupport into three South-Asian languages (Urdu, Punjabi and Bengali) to ensure minority ethnic groups in the UK can also access the support in a way that is culturally appropriate for them.

For peer review only - http://bmjopen.bmj.com/site/about/guidelines.xhtml

CONTRIBUTORS

GW leads the project and is responsible for study integrity. GW, JS, ZH, KE, PM-A, KAS, RTE, CJ, PB and GH critically reviewed the study proposal and secured the research funding. JS, AS and KE lead collaboration sites. PM-A leads workstreams 2 and 4. RTE leads workstream 3. GF manages the study and led the ethics submission. NG is the trial statistician. DS advised on quality assurance. GW, JS, ZH, KE, PM-A, KAS, RTE, CJ, PB, GH, NG and DS contributed to the research protocol, provided edits and critiqued the manuscript for intellectual content.

ACKNOWLEDGMENTS

The authors are extremely grateful to Professor Anne Magriet Pot, Erasmus School of Policy and Health Management, Erasmus University, the Netherlands for her inspiration and encouragement to seek funding for this project, the Caban Group for their thoughtful and useful insights, and the research assistants (Danielle Proctor, Gwenllian Hughes, John Connaghan, Fatene Abakar Ismail) for piloting the case report forms and to Stéfanie Freel and Katrin Seeher of the World Health Organisation for their comments on the funding application and ongoing support for the project delivery.

FUNDING

This project is funded by the National Institute for Health Research (NIHR) Public Health Research programme (project reference NIHR130914). The views expressed are those of the authors and not necessarily those of the NIHR or the Department of Health and Social Care.

DATA AVAILABILITY STATEMENT

The datasets generated during and/or analysed during the study will be available upon request via the corresponding author.

For peer review only - http://bmjopen.bmj.com/site/about/guidelines.xhtml

COMPETING INTERESTS

The authors declare they have no competing interests.

REFERENCES

[1] World Health Organization. Global status report on the public health response to dementia [Internet]. Geneva: World Health Organization; 2021 Sep [cited 2022 Jan 28]. https://www.who.int/publications/i/item/9789240033245

[2] Wittenberg R, Hu B, Barraza-Araiza L, et al. Projections of older people with dementia and costs of dementia care in the United Kingdom, 2019–2040: Working paper [Internet]. London: Care Policy and Evaluation Centre; 2019 Nov [cited 2022 Jan 28]. https://www.alzheimers.org.uk/sites/default/files/2019-11/cpec_report_november_2019.pdf

[3] Lewis F, Karlsberg S, Sussex J, et al. Trajectory of dementia in the UK – making a difference: consulting report [Internet]. London: Office of Health Economics; 2014 Jun [cited 2022 Jan 28]. https://www.alzheimersresearchuk.org/wp-content/uploads/2015/01/OHE-report-Full.pdf

[4] Gilhooly KJ, Gilhooly ML, Sullivan MP, et al. A meta-review of stress, coping and interventions in dementia and dementia caregiving. *BMC Geriatr*. 2016 May 18;16:106. doi: 10.1186/s12877-016-0280-8

[5] Bremer P, Cabrera E, Leino-Kilpi H, et al. Informal dementia care: Consequences for caregivers' health and health care use in 8 European countries. *Health Policy*. 2015 Nov;119(11):1459-71. doi: 10.1016/j.healthpol.2015.09.014 [published Online First: 22 October 2022].

[6] Pinquart M, Sörensen S. Differences between caregivers and noncaregivers in psychological health and physical health: a meta-analysis. *Psychol Aging*. 2003 Jun;18(2):250-67. doi: 10.1037/0882-7974.18.2.250

For peer review only - http://bmjopen.bmj.com/site/about/guidelines.xhtml

[7] McCabe M, You E, Tatangelo G. Hearing Their Voice: A Systematic Review of Dementia Family Caregivers' Needs. *Gerontologist*. 2016 Oct;56(5):e70-88. doi: 10.1093/geront/gnw078 [published Online First: 21 April 2016].

[8] World Health Organization. Global action plan on the public health response to dementia 2017–2025 [Internet]. Geneva: World Health Organization; 2017 [cited 2022 Jan 28]. https://apps.who.int/iris/bitstream/handle/10665/259615/9789241513487-eng.pdf?sequence=1

[9] Dickinson C, Dow J, Gibson G, et al. Psychosocial intervention for carers of people with dementia: What components are most effective and when? A systematic review of systematic reviews. *Int Psychogeriatr*. 2017 Jan;29(1):31-43. doi: 10.1017/S1041610216001447 [published Online First: 26 September 2016].

[10] Masterson-Algar P, Allen MC, Hyde M, et al. Exploring the impact of Covid-19 on the care and quality of life of people with dementia and their carers: A scoping review. *Dementia*. Published Online First: 1 November 2021. https://doi.org/10.1177/14713012211053971

[11] Egan KJ, Pinto-Bruno ÁC, Bighelli I, et al. Online Training and Support Programs Designed to Improve Mental Health and Reduce Burden Among Caregivers of People With Dementia: A Systematic Review. *J Am Med Dir Assoc*. 2018 Mar;19(3):200-206.e1. doi: 10.1016/j.jamda.2017.10.023 [published Online First: 4 January 2018].

[12] Godwin KM, Mills WL, Anderson JA, et al. Technology-driven interventions for caregivers of persons with dementia: a systematic review. *Am J Alzheimers Dis Other Demen*. 2013 May;28(3):216-22. doi: 10.1177/1533317513481091 [published Online First: 25 March 2013].

For peer review only

[13] Gitlin LN, Marx K, Stanley IH, et al. Translating Evidence-Based Dementia Caregiving Interventions into Practice: State-of-the-Science and Next Steps. *Gerontologist*. 2015 Apr;55(2):210-26. doi: 10.1093/geront/gnu123 [published Online First: 17 February 2015].

[14] Peterson K, Hahn H, Lee AJ, et al. In the Information Age, do dementia caregivers get the information they need? Semi-structured interviews to determine informal caregivers' education needs, barriers, and preferences. *BMC Geriatr*. 2016 Sep;16(164). doi:10.1186/s12877-016-0338-7

[15] Christie HL, Martin JL, Connor J, et al. eHealth interventions to support caregivers of people with dementia may be proven effective, but are they implementation-ready? *Internet Interv*. 2019 Jul;18:100260. doi: 10.1016/j.invent.2019.100260

[16] Hopwood J, Walker N, McDonagh L, et al. Internet-Based Interventions Aimed at Supporting Family Caregivers of People With Dementia: Systematic Review. *J Med Internet Res*. 2018 Jun;20(6):e216. doi: 10.2196/jmir.9548

[17] Pinto-Bruno ÁC, Pot AM, Kleiboer A, et al. An Online Minimally Guided Intervention to Support Family and Other Unpaid Carers of People With Dementia: Protocol for a Randomized Controlled Trial. *JMIR Res Protoc*. 2019 Oct;8(10):e14106. doi: 10.2196/14106

[18] Mehta KM, Gallagher-Thompson D, Varghese M, et al. iSupport, an online training and support program for caregivers of people with dementia: study protocol for a randomized controlled trial in India. *Trials*. 2018 May;19(1):271. doi: 10.1186/s13063-018-2604-9

[19] Teles S, Ferreira A, Seeher K, et al. Online training and support program (iSupport) for informal dementia caregivers: protocol for an intervention study in Portugal. *BMC Geriatr*. 2020 Jan;20(1):10. doi: 10.1186/s12877-019-1364-z

For peer review only - http://bmjopen.bmj.com/site/about/guidelines.xhtml

[20] Baker JR, Jeon YH, Goodenough B, et al. What do children need to know about dementia? The perspectives of children and people with personal experience of dementia. *Int Psychogeriatr*. 2018 May;30(5):673-684. doi: 10.1017/S1041610217002022 [published Online First: 2 October 2017].

[21] National Institute for Health and Care Excellence (NICE). Methods for the development of NICE public health guidance [Internet]. 3rd ed. UK: NICE; c2006 [updated 2012 Sep; cited 2022 Jan 28]. https://www.nice.org.uk/process/pmg4/resources/methods-for-the-development-of-nice-public-health-guidance-third-edition-pdf-2007967445701

[22] Chan AW, Tetzlaff JM, Altman DG, et al. SPIRIT 2013 statement: defining standard protocol items for clinical trials. *Ann Intern Med*. 2013 Feb;158(3):200-7. doi: 10.7326/0003-4819-158-3-201302050-00583

[23] Join Dementia Research is funded by the Department of Health and delivered by the National Institute for Health Research in partnership with Alzheimer Scotland, Alzheimer's Research UK and Alzheimer's Society. www.joindementiaresearch.nihr.ac.uk [accessed 10/03/2021].

[24] Deeken F, Rezo A, Hinz M, et al. Evaluation of Technology-Based Interventions for Informal Caregivers of Patients With Dementia-A Meta-Analysis of Randomized Controlled Trials. *Am J Geriatr Psychiatry*. 2019 Apr;27(4):426-445. doi: 10.1016/j.jagp.2018.12.003. Epub 2018 Dec 6. PMID: 30642650.

[25] Sugimoto T, Sozu T, Hamasaki T. A convenient formula for sample size calculations in clinical trials with multiple co-primary continuous endpoints. *Pharm Stat*. 2012 Mar-Apr;11(2):118-28. doi: 10.1002/pst.505

For peer review only - http://bmjopen.bmj.com/site/about/guidelines.xhtml

[26] Sozu T, Sugimoto T, Hamasaki T, et al. Sample Size Determination in Clinical Trials with Multiple Endpoints. Springer International Publishing; 2015. https://doi.org/10.1007/978-3-319-22005-5

[27] Russell D, Hoare ZS, Whitaker R, et al. Generalized method for adaptive randomization in clinical trials. *Stat Med*. 2011 Apr;30(9):922-34. doi: 10.1002/sim.4175 [published Online First: 1 February 2011].

[28] Sörensen S, Pinquart M, Duberstein P. How effective are interventions with caregivers? An updated meta-analysis. *Gerontologist*. 2002 Jun;42(3):356-72. doi: 10.1093/geront/42.3.356

[29] Kitwood T. Dementia reconsidered: The person comes first. UK: Open University Press; 1997.

[30] Pot AM, Gallagher-Thompson D, Xiao LD, et al. iSupport: a WHO global online intervention for informal caregivers of people with dementia. *World Psychiatry*. 2019 Oct;18(3):365-366. doi: 10.1002/wps.20684

[31] Office for National Statistics (ONS). 2011 Census: Key statistics for Wales, March 2011 [Internet]. UK: ONS; 2012 Dec. Section 10. Proficiency in Welsh; [cited 2022 Jan 28]; https://www.ons.gov.uk/peoplepopulationandcommunity/populationandmigration/populationestimates/bulletins/2011censuskeystatisticsforwales/2012-12-11#proficiency-in-welsh

[32] Alzheimer's Society. Caring for a person with dementia: A practical guide. London: Alzheimer's Society, 2002 [reviewed 2019]. Available from: https://www.alzheimers.org.uk/get-support/publications-factsheets/caring-person-dementia-practical-guide

[33] Elsevier Ltd. MACRO (Version 4.9.0.8594) [Computer software]. London; c2018

For peer review only - http://bmjopen.bmj.com/site/about/guidelines.xhtml

[34] Vasileiou K, Barnett J, Thorpe S, et al. Characterising and justifying sample size sufficiency in interview-based studies: systematic analysis of qualitative health research over a 15-year period. *BMC Med Res Methodol*. 2018 Nov;18(1):148. doi: 10.1186/s12874-018-0594-7

[35] Ritchie J, Lewis J, Elam G. Designing and selecting samples. In: Ritchie J, Lewis J, editors. Qualitative research practice: a guide for social science students and researchers. London: SAGE Publications Ltd.; 2003. p.77–108.

[36] Moore GF, Audrey S, Barker M, et al. Process evaluation of complex interventions: Medical Research Council guidance. *BMJ*. 2015 Mar;350:h1258. doi: 10.1136/bmj.h1258

[37] O'Cathain A, Croot L, Duncan E, et al. Guidance on how to develop complex interventions to improve health and healthcare. *BMJ Open*. 2019 Aug;9(8):e029954. doi: 10.1136/bmjopen-2019-029954

[38] May C, Finch T, Mair F, et al. Understanding the implementation of complex interventions in health care: the normalization process model. *BMC Health Serv Res*. 2007 Sep;7:148. doi: 10.1186/1472-6963-7-148

[39] Brooke J. SUS: A 'quick and dirty' usability scale. In: Jordan PW, Thomas B, Weerdmeester BA, McClelland IL, editors. Usability Evaluation in Industry. London: Taylor Francis; 1996:189–94.

[40] Lancaster GA, Dodd S, Williamson PR. Design and analysis of pilot studies: recommendations for good practice. *J Eval Clin Pract*. 2004 May;10(2):307-12. doi: 10.1111/j..2002.384.doc.x

[41] Braun V, Clarke V. Using thematic analysis in psychology. *Qual Res Psychol*. 2006;3(2): 77-101. doi: 10.1191/1478088706qp063oa

For peer review only - http://bmjopen.bmj.com/site/about/guidelines.xhtml

[42] Pfadenhauer LM, Gerhardus A, Mozygemba K, et al. Making sense of complexity in context and implementation: the Context and Implementation of Complex Interventions (CICI) framework. *Implement Sci*. 2017 Feb;12(1):21. doi: 10.1186/s13012-017-0552-5

[43] Fenwick E, O'Brien BJ, Briggs A. Cost-effectiveness acceptability curves--facts, fallacies and frequently asked questions. *Health Econ*. 2004 May;13(5):405-15. doi: 10.1002/hec.903

[44] Wiltsey Stirman S, Baumann AA, Miller CJ. The FRAME: an expanded framework for reporting adaptations and modifications to evidence-based interventions. *Implement Sci*. 2019 Jun;14(1):58. doi: 10.1186/s13012-019-0898-y

[45] Dementia Engagement and Empowerment Project (DEEP). The DEEP-Ethics Gold Standards for Dementia Research [Internet]. Version 1. Exeter (UK): Innovations in Dementia; 2020 [cited 2022 Jan 28]. https://www.dementiavoices.org.uk/wp-content/uploads/2020/07/The-DEEP-Ethics-Gold-Standards-for-Dementia-Research.pdf

[46] Eysenbach G; CONSORT-EHEALTH Group. CONSORT-EHEALTH: improving and standardizing evaluation reports of Web-based and mobile health interventions. *J Med Internet Res*. 2011 Dec;13(4):e126. doi: 10.2196/jmir.1923

[47] Husereau D, Drummond M, Augustovski F, et al. Consolidated Health Economic Evaluation Reporting Standards 2022 (CHEERS 2022) Statement: Updated Reporting Guidance for Health Economic Evaluations. *Value Health*. 2022 Jan;25(1):3-9. doi: 10.1016/j.jval.2021.11.1351

[i] Bédard M, Molloy DW, Squire L, et al. The Zarit Burden Interview: a new short version and screening version. *Gerontologist*. 2001 Oct;41(5):652-7. doi: 10.1093/geront/41.5.652.

For peer review only - http://bmjopen.bmj.com/site/about/guidelines.xhtml

[ii] Andresen EM, Byers K, Friary J, et al. Performance of the 10-item Center for Epidemiologic Studies Depression scale for caregiving research. *SAGE Open Med*. 2013 Dec;1:2050312113514576. doi: 10.1177/2050312113514576.

[iii] Herdman M, Gudex C, Lloyd A, et al. Development and preliminary testing of the new five-level version of EQ-5D (EQ-5D-5L). *Qual Life Res*. 2011 Dec;20(10):1727-36. doi: 10.1007/s11136-011-9903-x [published Online First: 09 April 2011].

[iv] Wagnild G. The Resilience Scale User's Guide for the US English version of the Resilience Scale and the 14-item Resilience Scale (RS-14). Worden, MT: The Resilience Center; 2009.

[v] Spitzer RL, Kroenke K, Williams JB, et al. A brief measure for assessing generalized anxiety disorder: the GAD-7. *Arch Intern Med*. 2006 May;166(10):1092-7. doi: 10.1001/archinte.166.10.1092.

[vi] Annear MJ, Toye C, Elliott KJ, et al. Dementia knowledge assessment scale (DKAS): confirmatory factor analysis and comparative subscale scores among an international cohort. *BMC Geriatr*. 2017 Jul;17(1):168. doi: 10.1186/s12877-017-0552-y.

[vii] Hoefman RJ, Van Exel NJA, Brouwer WBF. iMTA Valuation of Informal Care Questionnaire (iVICQ). Version 1.0 (December 2011). Rotterdam: iBMG / iMTA, 2011.

[viii] Spruytte N, Van Audenhove C, Lammertyn F. The Scale for the Quality of the Current Relationship. Internal Report. Leuven: LUCAS-KULeuven; 2000.

[ix] Mulhern B, Rowen D, Brazier J, et al. Development of DEMQOL-U and DEMQOL-PROXY-U: generation of preference-based indices from DEMQOL and DEMQOL-PROXY

For peer review only

For peer review only - http://bmjopen.bmj.com/site/about/guidelines.xhtml

for use in economic evaluation. *Health Technol Assess*. 2013 Feb;17(5):v-xv, 1-140. doi: 10.3310/hta17050.

Figures

Figure 1. iSupport content.

Figure 2. Recruitment flowchart.

For peer review only

For peer review only - http://bmjopen.bmj.com/site/about/guidelines.xhtml

Figure 1: The content of iSupport for dementia carers

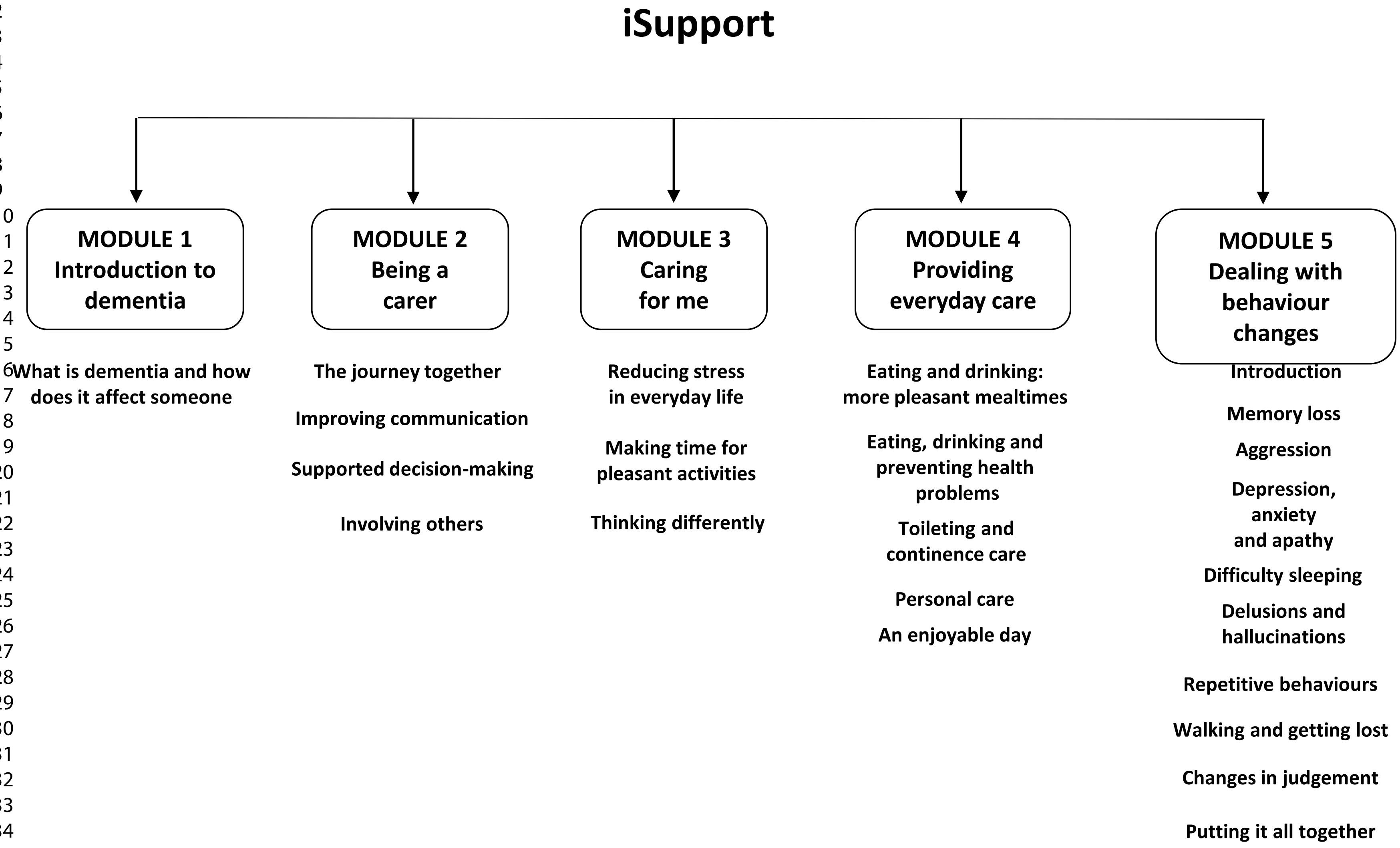

Figure 2: Recruitment flow chart

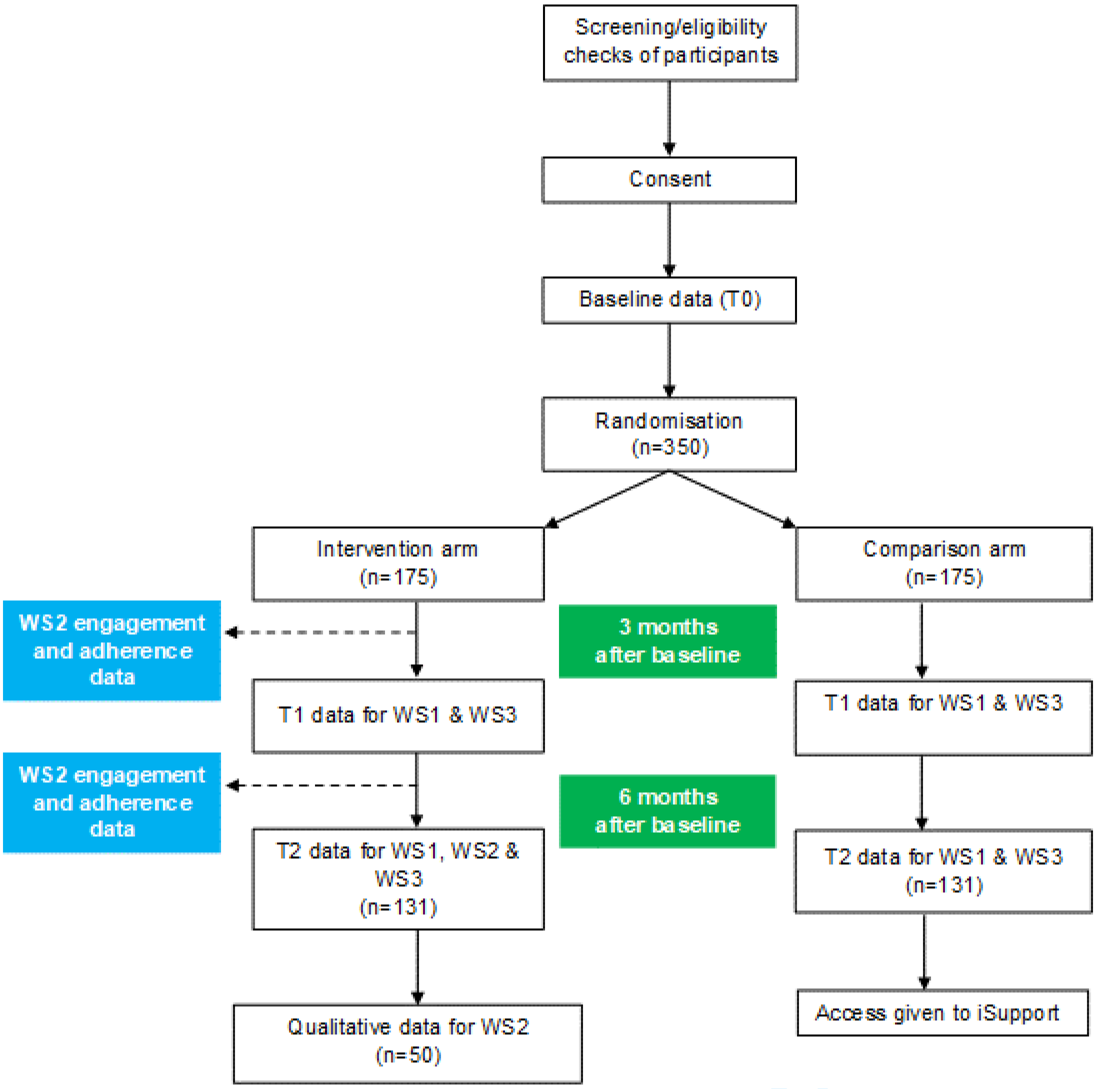

only

Supplementary Material

File 1 Objectives for each work-stream

WS1. A definitive pragmatic individually randomised controlled trial across Wales, Scotland and England, with a six-month nested internal pilot. This will:

• Determine progression of the definitive trial based on a go/review/stop criteria (nested internal pilot).
• Determine the effectiveness of 'iSupport' in reducing symptoms of distress and/or depression.

• Determine the effectiveness of 'iSupport' in reducing symptoms of anxiety.

• Determine the effectiveness of 'iSupport' in improving dementia knowledge, relationship quality and resilience.

• Describe the trial sample according to demographic/socioeconomic characteristics.

WS2. A process evaluation will be conducted in line with the established guidelines for process evaluations of complex evaluations[15,16] to determine the barriers and facilitators to the implementation of 'iSupport' at scale, and the extent it supports carers in the face of the ongoing or future COVID-19 pandemic. This will:
• Determine participant engagement and adherence to 'iSupport'.
• Explore the mechanisms of change.

• Identify the external factors to 'iSupport' which influence the delivery and function of the intervention.

• Explore the contextual factors that influence the scalability of 'iSupport' into wider contexts using the CICI framework.[17]

WS3. A parallel cost-effectiveness analysis, undertaken from both a public sector perspective (NHS, personal social services and local authorities), and a societal perspective (public sector plus opportunity costs). This will:
• Calculate the costs of implementing 'iSupport', including technical support and time spent supporting carers to use the tool.

• Explore patterns of, and estimate the cost of, health and social care resource use for carers in the 'iSupport' and comparison arms of the trial.

• Explore patterns of, and estimate the cost of, health and social care resource use for the care recipients of carers in the trial.

• Explore the opportunity cost of informal care through the measurement of informal care time, types of care task, impacts on carer's leisure and employment hours, and carers' willingness to pay for more support.

• Using QALYs derived from the EQ-5D-5L, determine the cost-effectiveness of 'iSupport' compared to the control condition; conduct secondary cost-effectiveness analyses using the Zarit Burden Interview[18] and the Centre for Epidemiological Studies of Depression Scale (CES-D10).[19,20]

For peer review only

File 2 Recruitment process

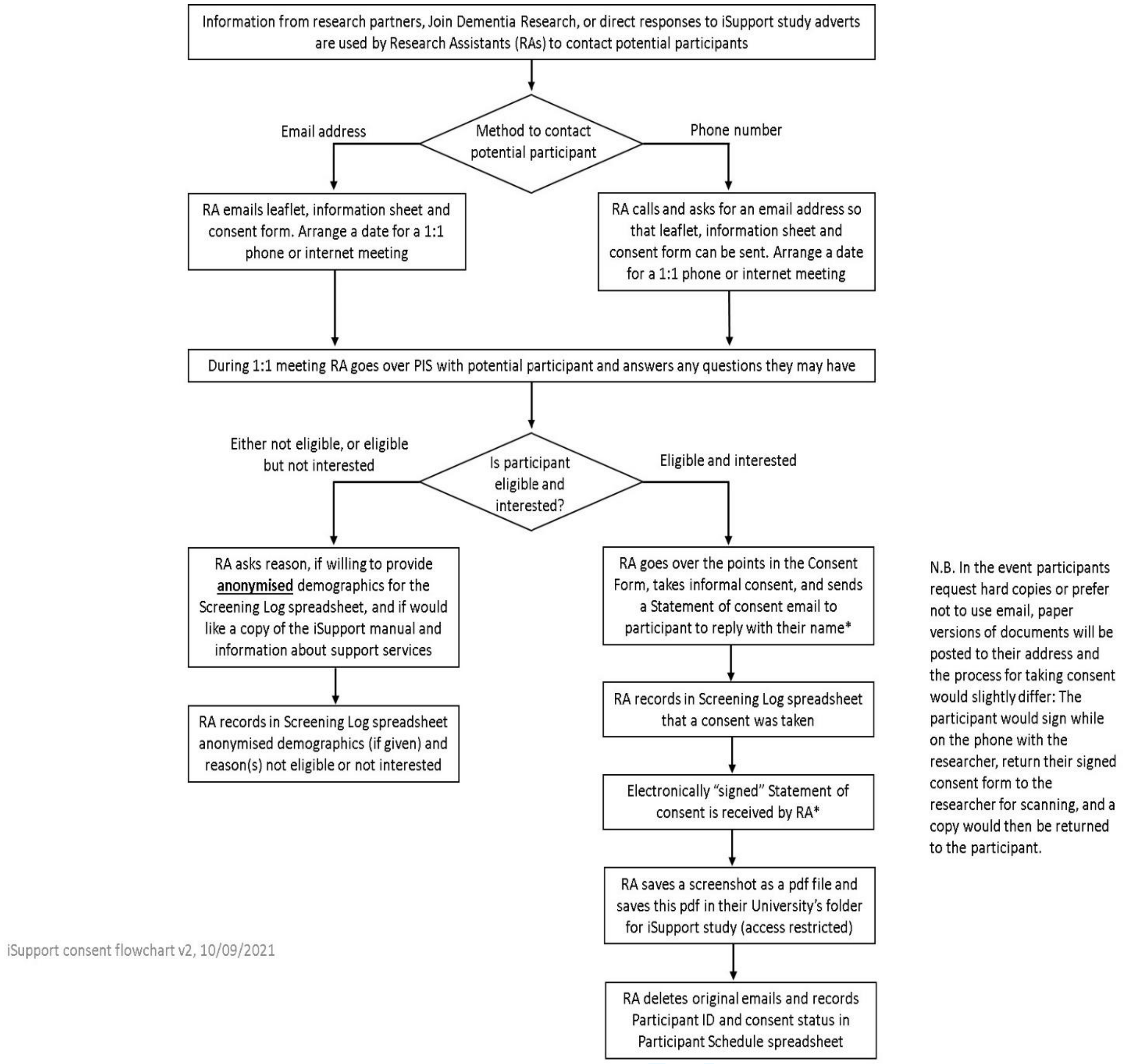

File 3 Feasibility study flowchart

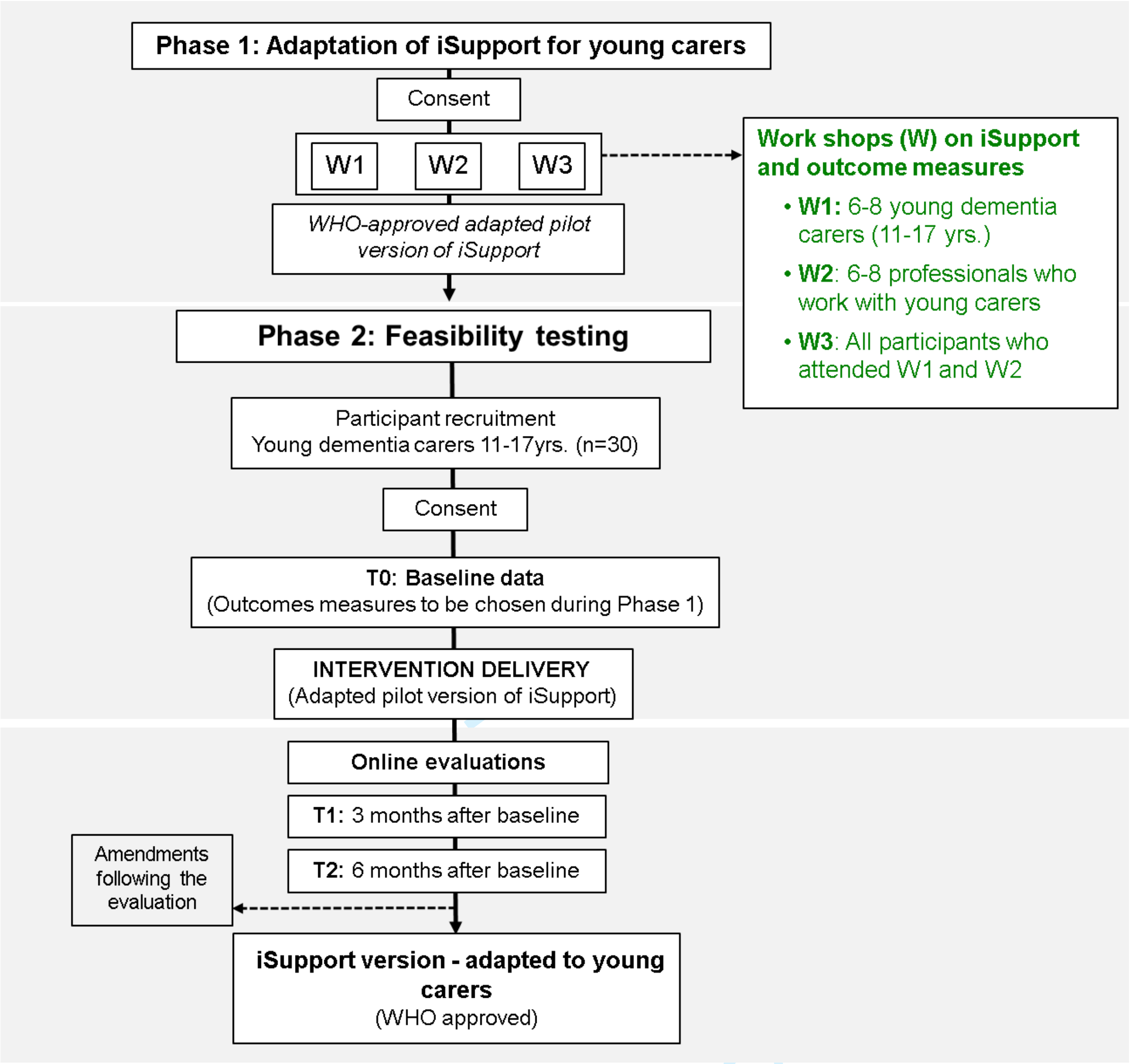

File 4 Consent Forms

iSupport for Dementia Carers – Main trial

Consent Form

Full title of project: A randomised controlled trial and feasibility study of the effects of an e-health intervention 'iSupport' for reducing distress of dementia carers, especially in the ongoing pandemic of COVID-19
Project number: NIHR_130914
Name of lead investigator: Prof. Gill Windle

[The process for technology-mediated consent is detailed in the protocol]

Participant identification number:________________

Please initial each box

1.	I confirm that I have read and understood the information sheet dated 25/10/2021 (version 2) for this study, had the opportunity to ask questions and have had these answered satisfactorily.	
2.	I understand that my participation is voluntary and that I am free to withdraw at any time, without giving any reason. I understand that if I withdraw this will not affect my health care or my legal rights in any way.	
3.	I understand that if I withdraw from the study the research team may continue to use the information that I previously provided up to that point, unless I indicate I do not want them to.	
4.	I understand that the information collected about me may be used to support other research in the future, and may be shared anonymously with other researchers.	
5.	I understand that I will not be identifiable in any data published in relation to this project.	
6.	I understand this study requires my involvement for six months and that I will be contacted by the research team approximately 3 months and 6 months after today's date.	
7.	I understand that if the researchers hear or observe anything that causes serious concern about my health, safety or well-being, or that of another person close to me, they have a duty to inform the lead investigator.	
8.	I agree that my anonymised data can be deposited and securely stored in a data archive.	
9.	I agree to take part in the above study.	

Name of Participant Date Signature

______________________ ______________ _____________

Name of person taking consent Date Signature

______________________ ______________ _____________

For peer review only

Discussing my experiences of using iSupport for Dementia Carers

Consent Form

Full title of project: A randomised controlled trial and feasibility study of the effects of an e-health intervention ‘iSupport’ for reducing distress of dementia carers, especially in the ongoing pandemic of COVID-19
Project number: NIHR_130914
Name of lead investigator: Prof. Gill Windle

[The process for technology-mediated consent is detailed in the protocol]

Participant identification number:________________

Please initial each box

1.	I confirm that I have read and understood the information sheet dated 25/10/2021 (version 2) for this part of the study, had the opportunity to ask questions and have had these answered satisfactorily.	
2.	I understand that my participation is voluntary and that I am free to withdraw at any time, without giving any reason. I understand that if I withdraw this will not affect my health care or my legal rights in any way.	
3.	I understand that if I withdraw from the study the research team may continue to use the information that I previously provided up to that point, unless I indicate I do not want them to.	
4.	I understand that the information collected about me may be used to support other research in the future, and may be shared anonymously with other researchers.	
5.	I agree to the interview being audio recorded as part of the research study.	
6.	I understand anonymised quotes from the interview may be included in any data published in relation to this project but that I will not be identifiable.	
7.	I understand that if the researchers hear or observe anything that causes serious concern about my health, safety or well-being, or that of another person close to me, they have a duty to inform the lead investigator.	
8.	I agree the anonymised transcripts and audio recordings of the interviews can be deposited and securely stored in a data archive.	
9.	I agree to take part in the above study.	

Name of Participant   Date   Signature

______________________   ______________   ______________

Name of person taking consent   Date   Signature

______________________   ______________   ______________

For peer review only

Adaptation of iSupport for younger dementia carers (Phase 1) Consent Form

Full title of project: A randomised controlled trial and feasibility study of the effects of an e-health intervention 'iSupport' for reducing distress of dementia carers, especially in the ongoing pandemic of COVID-19
Project number: NIHR_130914
Name of lead investigator: Prof. Gill Windle

[The process for technology-mediated consent is detailed in the protocol]

Participant identification number:_______________

Please initial each box

1.	I confirm that I have read and understood the information sheet dated 25/10/2021 (version 3) for this study, had the opportunity to ask questions and have had these answered satisfactorily.	
2.	I understand that my participation is voluntary and that I am free to withdraw at any time, without giving any reason. I understand that if I withdraw this will not affect my health care or my legal rights in any way.	
3.	I agree to the workshops being audio and video recorded by the researcher as part of the study.	
4.	I understand that if I withdraw from the study the research team may continue to use the information that I previously provided up to that point, unless I indicate I do not want them to.	
5.	I understand that the information collected about me may be used to support other research in the future, and may be shared anonymously with other researchers.	
6.	I understand that I will not be identifiable in any data published in relation to this project.	
7.	I agree that my anonymised data can be deposited and securely stored in a data archive.	
8.	I understand that if the researchers hear or observe anything that causes serious concern about my health, safety or well-being, or that of another person close to me, they have a duty to inform the lead investigator.	
9.	I understand that as part of the study there is a procedure in place which deals with disclosures of malpractice or abuse reported by participants and in such instances researchers will be required to break confidentiality.	
10.	I agree to take part in the above study.	

Name of Participant   Date   Signature

_______________   _______________   _______________

Name of person taking consent   Date   Signature

_______________   _______________   _______________

For peer review only

Feasibility testing iSupport for younger dementia carers (Phase 2) Consent Form

Full title of project: A randomised controlled trial and feasibility study of the effects of an e-health intervention 'iSupport' for reducing distress of dementia carers, especially in the ongoing pandemic of COVID-19
Project number: NIHR_130914
Name of lead investigator: Prof. Gill Windle

[The process for technology-mediated consent is detailed in the protocol]

Participant identification number:________________

Please initial each box

1.	I confirm that I have read and understood the information sheet dated 25/10/2021 (version 2) for this study, had the opportunity to ask questions and have had these answered satisfactorily.	
2.	I understand that my participation is voluntary and that I am free to withdraw at any time, without giving any reason. I understand that if I withdraw this will not affect my health care or my legal rights in any way.	
3.	I understand this study requires my involvement for six months and that I will be contacted by the research team approximately 3 months and 6 months after today's date.	
4.	I understand that if I withdraw from the study the research team may continue to use the information that I previously provided up to that point, unless I indicate I do not want them to.	
5.	I understand that the information collected about me may be used to support other research in the future, and may be shared anonymously with other researchers.	
6.	I understand that I will not be identifiable in any data published in relation to this project.	
7.	I agree that my anonymised data can be deposited and securely stored in a data archive.	
8.	I understand that if the researchers hear or observe anything that causes serious concern about my health, safety or well-being, or that of another person close to me, they have a duty to inform the lead investigator.	
9.	I understand that as part of the study there is a procedure in place which deals with disclosures of malpractice or abuse reported by participants and in such instances researchers will be required to break confidentiality.	
10.	I agree to take part in the above study.	

Name of Participant | Date | Signature

____________________ | ____________ | ____________

Name of person taking consent | Date | Signature

____________________ | ____________ | ____________

For peer review only

For peer review only

SPIRIT

STANDARD PROTOCOL ITEMS: RECOMMENDATIONS FOR INTERVENTIONAL TRIALS

SPIRIT 2013 Checklist: Recommended items to address in a clinical trial protocol and related documents*

	Section/item	Item No	Description
	**Administrative information**		
✓	Title	1	Descriptive title identifying the study design, population, interventions, and, if applicable, trial acronym
✓	Trial registration	2a	Trial identifier and registry name. If not yet registered, name of intended registry
✓		2b	All items from the World Health Organization Trial Registration Data Set
✓	Protocol version	3	Date and version identifier
✓	Funding	4	Sources and types of financial, material, and other support
✓	Roles and responsibilities	5a	Names, affiliations, and roles of protocol contributors
✓		5b	Name and contact information for the trial sponsor
✓		5c	Role of study sponsor and funders, if any, in study design; collection, management, analysis, and interpretation of data; writing of the report; and the decision to submit the report for publication, including whether they will have ultimate authority over any of these activities
✓		5d	Composition, roles, and responsibilities of the coordinating centre, steering committee, endpoint adjudication committee, data management team, and other individuals or groups overseeing the trial, if applicable (see Item 21a for data monitoring committee)
	**Introduction**		
✓	Background and rationale	6a	Description of research question and justification for undertaking the trial, including summary of relevant studies (published and unpublished) examining benefits and harms for each intervention
✓		6b	Explanation for choice of comparators
✓	Objectives	7	Specific objectives or hypotheses
✓	Trial design	8	Description of trial design including type of trial (eg, parallel group, crossover, factorial, single group), allocation ratio, and framework (eg, superiority, equivalence, noninferiority, exploratory)

For peer review only

Methods: Participants, interventions, and outcomes

✓	Study setting	9	Description of study settings (eg, community clinic, academic hospital) and list of countries where data will be collected. Reference to where list of study sites can be obtained
✓	Eligibility criteria	10	Inclusion and exclusion criteria for participants. If applicable, eligibility criteria for study centres and individuals who will perform the interventions (eg, surgeons, psychotherapists)
✓	Interventions	11a	Interventions for each group with sufficient detail to allow replication, including how and when they will be administered
✓		11b	Criteria for discontinuing or modifying allocated interventions for a given trial participant (eg, drug dose change in response to harms, participant request, or improving/worsening disease)
✓		11c	Strategies to improve adherence to intervention protocols, and any procedures for monitoring adherence (eg, drug tablet return, laboratory tests)
✓		11d	Relevant concomitant care and interventions that are permitted or prohibited during the trial
✓	Outcomes	12	Primary, secondary, and other outcomes, including the specific measurement variable (eg, systolic blood pressure), analysis metric (eg, change from baseline, final value, time to event), method of aggregation (eg, median, proportion), and time point for each outcome. Explanation of the clinical relevance of chosen efficacy and harm outcomes is strongly recommended
✓	Participant timeline	13	Time schedule of enrolment, interventions (including any run-ins and washouts), assessments, and visits for participants. A schematic diagram is highly recommended (see Figure)
✓	Sample size	14	Estimated number of participants needed to achieve study objectives and how it was determined, including clinical and statistical assumptions supporting any sample size calculations
✓	Recruitment	15	Strategies for achieving adequate participant enrolment to reach target sample size

Methods: Assignment of interventions (for controlled trials)

Allocation:

✓	Sequence generation	16a	Method of generating the allocation sequence (eg, computer-generated random numbers), and list of any factors for stratification. To reduce predictability of a random sequence, details of any planned restriction (eg, blocking) should be provided in a separate document that is unavailable to those who enrol participants or assign interventions

Allocation concealment mechanism	16b	Mechanism of implementing the allocation sequence (eg, central telephone; sequentially numbered, opaque, sealed envelopes), describing any steps to conceal the sequence until interventions are assigned
Implementation	16c	Who will generate the allocation sequence, who will enrol participants, and who will assign participants to interventions
Blinding (masking)	17a	Who will be blinded after assignment to interventions (eg, trial participants, care providers, outcome assessors, data analysts), and how
	17b	If blinded, circumstances under which unblinding is permissible, and procedure for revealing a participant's allocated intervention during the trial
**Methods: Data collection, management, and analysis**		
Data collection methods	18a	Plans for assessment and collection of outcome, baseline, and other trial data, including any related processes to promote data quality (eg, duplicate measurements, training of assessors) and a description of study instruments (eg, questionnaires, laboratory tests) along with their reliability and validity, if known. Reference to where data collection forms can be found, if not in the protocol
	18b	Plans to promote participant retention and complete follow-up, including list of any outcome data to be collected for participants who discontinue or deviate from intervention protocols
Data management	19	Plans for data entry, coding, security, and storage, including any related processes to promote data quality (eg, double data entry; range checks for data values). Reference to where details of data management procedures can be found, if not in the protocol
Statistical methods	20a	Statistical methods for analysing primary and secondary outcomes. Reference to where other details of the statistical analysis plan can be found, if not in the protocol
	20b	Methods for any additional analyses (eg, subgroup and adjusted analyses)
	20c	Definition of analysis population relating to protocol non-adherence (eg, as randomised analysis), and any statistical methods to handle missing data (eg, multiple imputation)
**Methods: Monitoring**		
Data monitoring	21a	Composition of data monitoring committee (DMC); summary of its role and reporting structure; statement of whether it is independent from the sponsor and competing interests; and reference to where further details about its charter can be found, if not in the protocol. Alternatively, an explanation of why a DMC is not needed

✓		21b	Description of any interim analyses and stopping guidelines, including who will have access to these interim results and make the final decision to terminate the trial
✓	Harms	22	Plans for collecting, assessing, reporting, and managing solicited and spontaneously reported adverse events and other unintended effects of trial interventions or trial conduct
✓	Auditing	23	Frequency and procedures for auditing trial conduct, if any, and whether the process will be independent from investigators and the sponsor
	**Ethics and dissemination**		
✓	Research ethics approval	24	Plans for seeking research ethics committee/institutional review board (REC/IRB) approval
✓	Protocol amendments	25	Plans for communicating important protocol modifications (eg, changes to eligibility criteria, outcomes, analyses) to relevant parties (eg, investigators, REC/IRBs, trial participants, trial registries, journals, regulators)
✓	Consent or assent	26a	Who will obtain informed consent or assent from potential trial participants or authorised surrogates, and how (see Item 32)
N/A		26b	Additional consent provisions for collection and use of participant data and biological specimens in ancillary studies, if applicable
✓	Confidentiality	27	How personal information about potential and enrolled participants will be collected, shared, and maintained in order to protect confidentiality before, during, and after the trial
✓	Declaration of interests	28	Financial and other competing interests for principal investigators for the overall trial and each study site
✓	Access to data	29	Statement of who will have access to the final trial dataset, and disclosure of contractual agreements that limit such access for investigators
N/A	Ancillary and post-trial care	30	Provisions, if any, for ancillary and post-trial care, and for compensation to those who suffer harm from trial participation
✓	Dissemination policy	31a	Plans for investigators and sponsor to communicate trial results to participants, healthcare professionals, the public, and other relevant groups (eg, via publication, reporting in results databases, or other data sharing arrangements), including any publication restrictions
✓		31b	Authorship eligibility guidelines and any intended use of professional writers
✓		31c	Plans, if any, for granting public access to the full protocol, participant-level dataset, and statistical code

For peer review only

Appendices

N/A	Informed consent materials	32	Model consent form and other related documentation given to participants and authorised surrogates
N/A	Biological specimens	33	Plans for collection, laboratory evaluation, and storage of biological specimens for genetic or molecular analysis in the current trial and for future use in ancillary studies, if applicable

*It is strongly recommended that this checklist be read in conjunction with the SPIRIT 2013 Explanation & Elaboration for important clarification on the items. Amendments to the protocol should be tracked and dated. The SPIRIT checklist is copyrighted by the SPIRIT Group under the Creative Commons "Attribution-NonCommercial-NoDerivs 3.0 Unported" license.

For peer review only
