# Supplementary material for: Effects of an e-health intervention ‘iSupport’ for reducing distress of dementia carers: protocol for a randomised controlled trial and feasibility study
Source: BMJ Open. 2022 Sep 21;12(9):e064314. doi: 10.1136/bmjopen-2022-064314 (PMC9494593; doi:10.1136/bmjopen-2022-064314)
Supplement: Reviewer comments [file bmjopen-2022-064314.reviewer_comments.pdf]

PEER REVIEW HISTORY

BMJ Open publishes all reviews undertaken for accepted manuscripts. Reviewers are asked to complete a checklist review form (**http://bmjopen.bmj.com/site/about/resources/checklist.pdf**) and are provided with free text boxes to elaborate on their assessment. These free text comments are reproduced below.

ARTICLE DETAILS

**TITLE (PROVISIONAL)**	The effects of an e-health intervention ('iSupport') for reducing distress of dementia carers: protocol for a randomised controlled trial and feasibility study
**AUTHORS**	Windle, Gill; Flynn, Greg; Hoare, Zoe; Masterson-Algar, Patricia; Egan, Kieren; Edwards, Rhiannon; Jones, Carys; Spector, Aimee; Algar-Skaife, Katherine; Hughes, Gwenllian; Brocklehurst, P; Goulden, Nia; Skelhorn, Debbie; Stott, Joshua

This article was not externally reviewed at BMJ Open. Protocols that have been independently assessed prior to submission to BMJ Open are usually fast-tracked to publication on the grounds that further substantial changes will not be possible. This independent assessment will usually be external, independent review for both a substantial grant award from a non-commercial or government funder and ethics approval.
